# Multidimensional Characterization of Surface Properties and Microbial Biofilm Formation in Commercial Flowable Resin-Based Dental Composites

**DOI:** 10.3390/jfb17080413

**Published:** 2026-08-18

**Authors:** Lucian Floare, Otilia Cornelia Bolos, Ramona Dumitrescu, Marioara Nicoleta Caraba, Iasmina-Mădălina Petculescu, Carmen Opris, Octavia Balean, Vanessa Bolchis, Bianca Ioana Todor, Cristina Elena Savencu, Atena Galuscan, Daniela Jumanca

**Affiliations:** 1 Translational and Experimental Clinical Research Centre in Oral Health, Clinic of Preventive, Community Dentistry and Oral Health, University of Medicine and Pharmacy “Victor Babes”, 300040 Timisoara, Romania; lucian.floare@umft.ro (L.F.); balean.octavia@umft.ro (O.B.); vanessa.bolchis@umft.ro (V.B.); galuscan.atena@umft.ro (A.G.); jumanca.daniela@umft.ro (D.J.); 2 Clinic of Preventive, Community Dentistry and Oral Health, Department I, University of Medicine and Pharmacy “Victor Babes”, Eftimie Murgu Sq. no 2, 300041 Timisoara, Romania; 3 Department of Dento-Facial Aesthetics, Faculty of Dental Medicine, University of Medicine and Pharmacy “Victor Babes”, 300041 Timisoara, Romania; bolos.otilia@umft.ro; 4 Department of Cellular and Molecular Biology, Faculty of Medicine, University of Medicine and Pharmacy “Victor Babes”, 300041 Timisoara, Romania; 5 ANAPATMOL Research Center, University of Medicine and Pharmacy “Victor Babes”, 300041 Timisoara, Romania; 6 Department of Materials Engineering and Manufacturing, Faculty of Mechanics, Politehnica University, 300222 Timisoara, Romania; iasmina.petculescu@upt.ro (I.-M.P.); carmen.opris@upt.ro (C.O.); 7 Department of Dentistry, Faculty of Medicine and Pharmacy, University of Oradea, 10 Piața 1 Decembrie Street, 410073 Oradea, Romania; biancaioana.todor@gmail.com; 8 Department of Dental Prostheses Technology, Faculty of Dentistry, University of Medicine and Pharmacy “Victor Babes”, 9 Revolutiei 1989 Blv., 300041 Timisoara, Romania; cristina.savencu@umft.ro

**Keywords:** composite resins, surface roughness, hardness, surface properties, biofilm, *Streptococcus mutans*, *Candida albicans*

## Abstract

The interaction between restorative materials and microbial biofilms plays an important role in restoration longevity and the development of secondary caries. This study aimed to perform a multidimensional characterization of four commercially available flowable resin-based dental composites by evaluating their surface roughness, Vickers microhardness, surface morphology, and microbial biofilm formation under standardized in vitro conditions. Filtek™ Bulk Fill Flowable Restorative (3M), Tetric EvoFlow (Ivoclar Vivadent), BRILLIANT Flow (Coltene), and G-ænial™ Universal Injectable (GC) were investigated. Surface roughness was determined by contact profilometry, microhardness was assessed using the Vickers method, and microstructural characteristics were analyzed by scanning electron microscopy (SEM). Microbial biofilm biomass formed by Gram-positive bacteria, Gram-negative bacteria, and *Candida albicans*, including both reference strains and clinical isolates, was quantified using a crystal violet assay to compare material-dependent microbial colonization. G-ænial™ Universal Injectable exhibited the lowest roughness values, whereas BRILLIANT Flow showed the highest microhardness. SEM analysis revealed a more homogeneous surface morphology for Filtek™ Bulk Fill Flowable Restorative and G-ænial™ Universal Injectable, while Tetric EvoFlow and BRILLIANT Flow displayed increased topographical heterogeneity. Although modest differences in biofilm biomass accumulation were observed among the investigated composites (4.37–12.21% relative biomass reduction compared with the control), no material demonstrated a distinct advantage under the experimental conditions. The integrated evaluation of surface roughness, microhardness, surface morphology, and microbial biofilm formation provides a comparative characterization of commercially available flowable resin-based composites and contributes to a better understanding of material–biofilm interactions. These findings may support the future development of multifunctional restorative biomaterials with improved biofilm-modulating properties.

## 1. Introduction

In recent years, resin-based dental materials have gained increasing importance in dental medicine due to their versatility and capacity to combine mechanical performance with biological functionality [1]. Among these, resin-based composites are widely used in restorative dentistry, with flowable variants being particularly valued for their enhanced handling characteristics and ability to adapt effectively to cavity walls. Their improved flowability and mechanical performance facilitate precise placement, especially in areas with complex geometries. Owing to these properties, flowable composites are extensively applied in various clinical procedures, including preventive restorations, the treatment of cervical lesions, restorations in primary teeth, cavity lining, and pit and fissure sealing [2]. However, several studies have reported that resin-based composites may exhibit a higher tendency for plaque accumulation and biofilm formation on their surfaces compared to natural tooth structures and other restorative materials [3,4]. This phenomenon is frequently observed at the interface between the restoration and the tooth, where it contributes to the development of secondary caries, ultimately compromising the longevity of restorations and leading to material failure [5,6]. This limitation is related to the intrinsic susceptibility of resin-based composites to biofilm accumulation, which represents a key factor in restoration failure [7]. While oral hygiene and dietary control remain essential strategies for preventing biofilm accumulation, differences in the physicochemical surface characteristics of restorative materials may also influence the initial stages of microbial adhesion and subsequent biofilm development. Therefore, comparative investigations of commercially available restorative materials are valuable for improving our understanding of material–biofilm interactions [8,9].

The oral cavity hosts a complex microbiota capable of forming structured biofilms on dental surfaces and restorative materials [10,11]. The accumulation of microbial biofilms is a key factor in the development of secondary caries, periodontal diseases, and persistent oral infections [12]. In particular, cariogenic bacteria such as *Streptococcus mutans* and *Streptococcus sanguinis*, as well as opportunistic pathogens like *Staphylococcus aureus* and *Candida albicans*, are known to colonize restorative surfaces and contribute to treatment failure [13]. Dental biofilms are highly organized three-dimensional microbial communities embedded within an extracellular polymeric substance (EPS) matrix composed of polysaccharides, proteins, nucleic acids, and lipids. This matrix plays a crucial role in microbial adhesion, structural integrity, intercellular communication, and survival, while also providing enhanced resistance to antimicrobial agents and environmental stressors [14]. Consequently, understanding how restorative materials influence microbial adhesion and biofilm formation has become an important consideration in the characterization of restorative biomaterials and in the development of future multifunctional restorative systems [15,16].

Polymeric dental composites, typically based on dimethacrylate monomers such as Bis-GMA, UDMA, and TEGDMA, combined with inorganic fillers, represent complex multifunctional systems [7]. Variations in resin matrix composition, filler type, particle size, and filler loading can influence not only mechanical and aesthetic properties but also surface characteristics that affect microbial adhesion and biofilm development [17]. Consequently, these materials can act as functional interfaces between the host tissue and the oral microbiota. Although conventional commercially available resin composites are not designed to exert intrinsic antibacterial activity, variations in their composition and surface characteristics may influence their interaction with microbial biofilms, thereby providing valuable comparative information for the future development of multifunctional restorative materials [7].

Surface characteristics play a crucial role in determining the clinical performance and biological behavior of resin-based composites. Parameters such as surface roughness, microhardness, and microstructural organization influence not only wear resistance and mechanical stability but also microbial adhesion and biofilm maturation. In addition, differences in filler distribution and resin matrix composition may contribute to variations in surface topography and material–microbe interactions [18,19,20]. Therefore, the combined evaluation of physicochemical and biological properties is essential for a comprehensive characterization of restorative materials.

Recent research has increasingly focused on understanding how the physicochemical characteristics of restorative materials influence microbial adhesion and biofilm formation. Particular attention has been directed toward surface roughness, filler architecture, resin matrix composition, and surface morphology, as these parameters may affect the initial stages of microbial colonization and subsequent biofilm maturation [7]. Although considerable progress has been achieved in the development of experimental bioactive and antibacterial restorative materials, comprehensive comparative evaluations of commercially available flowable resin composites integrating physicochemical surface characterization with quantitative biofilm assessment remain comparatively underrepresented in the literature. Most previous studies have focused on individual physicochemical or mechanical properties, whereas integrated characterization of surface properties together with microbial biofilm formation provides a more comprehensive understanding of material–biofilm interactions and may support the future development of multifunctional restorative biomaterials.

Although the physicochemical and mechanical properties of contemporary flowable resin-based composites have been extensively investigated, comparatively fewer studies have integrated standardized physicochemical surface characterization with microbial biofilm assessment within the same experimental framework. Accordingly, the present study was designed as a biomaterials investigation to characterize material–biofilm interactions under standardized laboratory conditions rather than to evaluate the intrinsic antibacterial properties of conventional restorative materials.

Given the complexity of the oral biofilm ecosystem, the present investigation was intentionally designed as a standardized in vitro laboratory study to enable reproducible comparisons among commercially available flowable resin-based composites. Accordingly, the findings should be interpreted within the context of this experimental model.

Therefore, the aim of the present study was to perform a multidimensional characterization of four commercially available flowable resin composites by integrating surface roughness, Vickers microhardness, surface morphology, and quantitative assessment of microbial biofilm biomass formed by a panel of reference strains and clinically relevant microbial isolates selected to provide a standardized comparative evaluation of material–biofilm interactions under in vitro conditions. The study was designed to explore whether differences in physicochemical surface characteristics are associated with distinct patterns of microbial biofilm formation. This integrated comparative approach provides a comprehensive characterization of commercially available flowable resin-based composites and may support the future development and evaluation of advanced multifunctional restorative biomaterials using progressively more biologically relevant biofilm models.

## 2. Materials and Methods

### 2.1. Materials

Four commercially available flowable dental resin composites were selected for this study based on their widespread clinical use and differences in composition and filler characteristics. The investigated materials included Filtek™ Bulk Fill Flowable Restorative (3M, St. Paul, MN, USA), Tetric EvoFlow (Ivoclar Vivadent, AG, Schaan, Liechtenstein), BRILLIANT Flow (Coltene/Whaledent AG, Altstätten, Switzerland), and G-ænial™ Universal Injectable (GC, Tokyo, Japan). These materials are frequently used in routine restorative procedures and were chosen as representative examples of contemporary bulk-fill, nanohybrid, and highly injectable flowable composites. Their formulations are based on dimethacrylate resin systems, including bisphenol A glycidyl methacrylate (Bis-GMA), urethane dimethacrylate (UDMA), D3MA (decanediol dimethacrylate), TEGDMA (triethylene glycol dimethacrylate), and proprietary dimethacrylate monomers, depending on the manufacturer composition (Table 1).

The investigated materials differ in resin matrix composition, filler architecture, filler loading, particle size distribution, and handling characteristics, representing distinct categories of contemporary flowable resin-based composites. In addition to their different clinical indications, these compositional and structural variations may influence surface morphology, roughness, mechanical behavior, and microbial adhesion. Therefore, these materials provide suitable models for investigating the relationship between surface properties and microbial biofilm formation. The selection criteria were based on their clinical relevance and their representativeness with respect to compositional and structural diversity, rather than on manufacturers’ claims regarding antibacterial activity or bioactive performance. Furthermore, the objective of the present study was not to reproduce isolated material characteristics described in manufacturer technical documentation or previous studies, but to evaluate all investigated materials under identical experimental conditions using a standardized and integrated characterization protocol. The present study intentionally focused on widely used conventional flowable resin-based composites rather than materials specifically designed with antimicrobial or bioactive properties, in order to evaluate the intrinsic influence of composition and surface characteristics on microbial biofilm formation under standardized in vitro conditions.

### 2.2. Specimen Preparation

For surface characterization analyses, including surface roughness, Vickers microhardness, and scanning electron microscopy (SEM), standardized disk-shaped specimens were fabricated from each investigated composite resin. A total of 40 specimens were prepared (10 specimens per material) using calibrated plexiglass molds with dimensions of 10 mm in diameter and 2 mm in thickness. The composite materials were packed into the molds positioned on a glass slab. A transparent Mylar strip was placed over the material surface, followed by a glass slide to obtain flat and standardized surfaces and to minimize the oxygen-inhibited layer. Gentle pressure was applied to remove excess material and prevent void formation. Polymerization was performed using a LED light-curing unit (Bluephase G2, Ivoclar Vivadent AG, Schaan, Liechtenstein) with an output intensity of 1200 mW/cm^2^. The curing tip was positioned approximately 1 mm from the specimen surface and perpendicular to the mold. Each specimen was light-cured for 20 s on both sides, according to the manufacturers’ recommendations and previously reported protocols for flowable resin-based composites. The light intensity of the curing unit was verified using a calibrated radiometer (Bluephase Meter II, Ivoclar Vivadent AG, FL-9494, Schaan, Liechtenstein) before specimen fabrication and periodically throughout the experimental procedures to ensure consistent light output. Following polymerization, the specimens were carefully removed from the molds, visually inspected for defects, and stored under standardized laboratory conditions prior to polishing and surface characterization analyses. The dimensions of all specimens were verified after removal from the molds to confirm conformity with the standardized mold dimensions (10 mm diameter × 2 mm thickness). In addition, all specimens were visually inspected by the same operator to verify the absence of voids, marginal defects, or surface irregularities prior to inclusion in the experimental analyses. Specimens exhibiting visible defects, air bubbles, or surface irregularities were excluded and replaced to ensure specimen homogeneity and reproducibility of the subsequent analyses.

### 2.3. Polishing Procedure

Following polymerization, all specimens underwent a standardized finishing and polishing procedure to obtain clinically relevant surface conditions and ensure consistency among groups. Polishing was performed using a sequential aluminum oxide disc system (Sof-Lex™, 3M ESPE Dental Products, St. Paul, MN, USA) under dry conditions with a low-speed handpiece operating at approximately 15,000 rpm. Each specimen was polished for 20 s with each disc in the recommended sequence: coarse (100 μm), medium (29 μm), fine (14 μm), and superfine (8 μm). A single operator performed all polishing procedures to minimize operator-dependent variability. New polishing discs were used for each specimen to avoid inconsistencies related to abrasive wear. After polishing, the specimens were ultrasonically cleaned in distilled water for 5 min to remove residual debris and abrasive particles. Subsequently, the samples were rinsed with distilled water and air-dried prior to surface roughness, microhardness, and SEM analyses. This standardized polishing protocol was adopted to provide uniform surface conditions and to minimize the influence of surface preparation on the evaluated properties. Polishing was performed exclusively on specimens intended for surface roughness, microhardness, and SEM analyses. Specimens used for biofilm assays were prepared directly in 96-well plates and were not polished in order to preserve their as-cured surface characteristics. Accordingly, biofilm formation was evaluated on the original polymerized composite surfaces.

### 2.4. Surface Roughness Analysis

Surface roughness measurements were performed using a contact profilometer (Surftest SJ-201, Mitutoyo Europe GmbH, Neuss, Germany) to characterize the surface topography of the investigated composites. The arithmetic average roughness (Ra, μm) was selected as the surface roughness parameter, as it represents one of the most commonly used indicators for evaluating the surface quality of resin-based restorative materials. The profilometer was equipped with a diamond stylus with a tip radius of 5 μm. Measurements were performed at a tracing speed of 0.5 mm/s, using a cutoff length of 0.8 mm and an evaluation length of 4 mm. For each specimen, three measurements were recorded at different locations on the polished surface, and the mean value was calculated and expressed as the Ra. All measurements were performed under standardized conditions to minimize experimental variability and ensure reproducibility. Surface roughness evaluation was performed according to previously described methodologies for resin-based dental materials [21,22].

### 2.5. Vickers Microhardness Analysis

Surface microhardness was evaluated using the Vickers indentation method with a microhardness tester (402MVD, Wolpert Group, Ludwigshafen, Germany). Measurements were performed using a diamond pyramid indenter under a load of 50 g applied for 10 s. Prior to testing, the specimens were securely positioned to ensure stable and reproducible measurements. To account for surface heterogeneity and improve measurement reliability, three indentations were performed at different locations on each specimen, avoiding the specimen margins and maintaining sufficient spacing between adjacent indentations. The diagonal lengths of the indentations were measured, and the corresponding Vickers hardness values were automatically calculated by the instrument and expressed as Vickers hardness number (HV). The mean value obtained from the three measurements was considered the final microhardness value for each specimen. All measurements were performed under standardized conditions to minimize experimental variability and ensure reproducibility. The Vickers indentation method was selected because of its suitability for evaluating the surface mechanical properties of resin-based dental composites and its widespread use in dental materials research.

### 2.6. Scanning Electron Microscopy (SEM) Analysis

Surface morphology of the investigated resin composites was evaluated by scanning electron microscopy (SEM) using a FEI Inspect S microscope (FEI Company, Eindhoven, The Netherlands). Representative specimens from each material group were mounted on aluminum stubs and coated with a thin conductive gold layer by plasma sputtering to minimize charging effects and improve image quality during electron beam examination.

SEM observations were performed under vacuum conditions at different magnifications ranging from ×1000 to ×25,000 in order to qualitatively evaluate surface morphology, topographical irregularities, and the exposure pattern and distribution of filler particles. Low magnifications were used to assess the overall surface appearance, whereas higher magnifications enabled detailed examination of filler architecture and surface microstructural features. The SEM images were used for qualitative comparison among materials and to support the interpretation of the roughness, microhardness, and microbial biofilm formation results.

### 2.7. Microbial Strains and Culture Conditions for Biofilm Assays

The biofilm assays were conducted using a diverse panel of Gram-positive and Gram-negative bacterial strains, along with a representative fungal strain, to provide a broader assessment of microbial responses to the tested materials. Both reference strains (American Type Culture Collection, ATCC, Manassas, VA, USA) and clinical isolates obtained from patients were included to account for microbial variability and better simulate clinically relevant conditions. The selected microorganisms were intended to represent different microbial groups commonly involved in biofilm-associated oral and opportunistic infections and to provide a standardized experimental model for evaluating material–biofilm interactions rather than reproducing the complexity of mature polymicrobial oral biofilms. Accordingly, a standardized monospecies static biofilm model was selected to minimize biological variability and enable reproducible comparisons among restorative materials under identical experimental conditions.

The Gram-positive bacterial strains comprised *Staphylococcus aureus* MRSA (ATCC 43300), a methicillin-resistant *Staphylococcus aureus* (MRSA) clinical isolate, *Staphylococcus aureus* (ATCC 29213), a clinical isolate of *Staphylococcus aureus*, *Staphylococcus epidermidis* (ATCC 14990), *Streptococcus sanguinis* (ATCC 10556), *Streptococcus mutans* (ATCC 35668), and *Lacticaseibacillus casei* (ATCC 393). Gram-negative bacterial strains included *Escherichia coli* (ATCC 25922) and a clinical isolate of *E. coli*, while *Candida albicans* (ATCC 10231) was included as a representative fungal species commonly associated with oral biofilm-related infections.

Reference strains were obtained from the culture collection of the Department of Cellular and Molecular Biology, “Victor Babeș” University of Medicine and Pharmacy, Timișoara, Romania. Clinical isolates were provided by the Microbiology Laboratory of the “Pius Brînzeu” Emergency Clinical County Hospital, Timișoara, Romania.

All bacterial strains were cultivated in Tryptic Soy Broth (TSB, Merck KGaA, Darmstadt, Germany) at 37 °C for 24 h under aerobic conditions. The fungal strain was cultivated in Sabouraud broth (Merck KGaA, Darmstadt, Germany) at 37 °C for 48 h. Following incubation, the optical density (OD) of each microbial suspension was measured at 600 nm using a microplate reader (BioTek Synergy H1, Agilent Technologies, Santa Clara, CA, USA) to ensure consistency in microbial growth. The cultures were adjusted to the logarithmic growth phase prior to use in biofilm assays. A standardized monospecies static biofilm model was intentionally selected to minimize biological variability and ensure reproducible comparison among restorative materials under identical experimental conditions. Although this model does not reproduce the complexity of the oral microbiome, it provides a controlled experimental platform suitable for comparative laboratory evaluation.

Subsequently, microbial suspensions were standardized to a turbidity equivalent to the 0.5 McFarland standard (approximately 1.5 × 10^8^ CFU/mL) using a McFarland densitometer (Grand-Bio, London, UK). The resulting standardized inocula were used in all subsequent experiments to ensure reproducibility and comparability among experimental groups.

### 2.8. Biofilm Formation and Biomass Quantification Assay

Microbial biofilm formation on the surface of the investigated flowable resin-based composites was evaluated using a modified crystal violet staining assay, based on the method described by Knezevic and Petrovic (2008) [23] and further adapted in recent studies [24,25,26]. This spectrophotometric method is widely used for the quantification of total biofilm biomass. The assay provides a relative assessment of adherent biofilm biomass and represents a simplified in vitro model for investigating material–biofilm interactions under standardized conditions. For biofilm experiments, approximately 60 μL of each composite material was aseptically dispensed into sterile 96-well microplates (Thermo Fisher Scientific, Kamstrupvej 91, 4000 Roskilde, Denmark), corresponding to a standardized thickness of approximately 2 mm. The specimens were photopolymerized for 20 s using a LED curing unit (Bluephase G2, Ivoclar Vivadent AG, Schaan, Liechtenstein) according to the manufacturers’ recommendations and maintained under aseptic conditions prior to microbial exposure. After polymerization, the specimens were visually inspected to confirm the absence of voids or surface defects and to ensure uniform specimen geometry prior to microbial exposure. No finishing or polishing procedures were performed prior to microbial inoculation, thereby preserving the original as-cured surface characteristics of the materials. The polymerized specimens represented the experimental material groups used for biofilm formation assays.

Control wells containing only microbial suspension without restorative material were used as positive controls and represented maximum biofilm formation. Additional wells containing polymerized materials without microbial inoculum were included to account for background absorbance, and the corresponding values were subtracted from the experimental readings.

Subsequently, 100 μL of standardized microbial suspension (0.5 McFarland) prepared in Tryptic Soy Broth (for bacterial strains) or Sabouraud broth (for the fungal strain) was added to each well. The microplates were incubated at 37 °C for 24 h in the case of bacterial strains and for 48 h in the case of *Candida albicans*, allowing biofilm formation on the surface of the tested materials. Incubation was performed under aerobic conditions.

Following incubation, non-adherent cells were carefully removed by washing the wells twice with sterile 0.9% NaCl solution. The plates were subsequently dried at 37 °C to allow fixation of the adherent biofilm biomass. Biofilms were stained with 200 μL of 0.4% crystal violet solution and incubated for 1 h at 37 °C. Excess stain was removed by thorough washing with distilled water. The bound dye, corresponding to the total biofilm biomass, was subsequently solubilized using 200 μL of 30% acetic acid and incubated for 30 min at room temperature. The absorbance of the resulting solutions was measured at 570 nm using a microplate reader (BioTek Synergy H1, Agilent Technologies, Santa Clara, CA, USA).

All measurements were performed in triplicate (technical replicates), and the entire experiment was independently repeated three times (biological replicates) to ensure reproducibility.

Relative biofilm biomass reduction (%) was calculated according to the following equation:

$$\mathrm{Relative}\;\mathrm{biofilm}\;\mathrm{biomass}\;\mathrm{reduction}\begin{array}{c} \%=\left[\frac{\left({\mathrm{OD}}_{\mathrm{control}}-{\mathrm{OD}}_{\mathrm{Sample}}\right)}{{\mathrm{OD}}_{\mathrm{control}}}\right]\times 100. \end{array}$$

where OD__sample_ represents the optical density of the biofilm formed in the presence of the tested material, and OD__control_ represents the optical density of the untreated control.

Control wells containing only microbial suspension without restorative material were considered to represent 100% biofilm formation and served as the reference condition for calculating the relative biofilm biomass reduction. Accordingly, a value of 0% relative biofilm biomass reduction indicates biofilm biomass comparable to that observed in the positive control rather than complete surface saturation. Because the crystal violet assay quantifies total biomass, the obtained values represent relative differences in biofilm biomass and should not be interpreted as absolute surface coverage or as a direct reproduction of the complexity of natural oral biofilms.

### 2.9. Statistical Analysis

All experimental data are presented as mean ± standard deviation (SD). Surface roughness and Vickers microhardness measurements were obtained from ten specimens per material, whereas biofilm assays were performed in triplicate and independently repeated three times. Statistical analysis was performed using IBM SPSS Statistics software (version 30.0, IBM Corp., Armonk, NY, USA). Prior to inferential analysis, the normality of data distribution was assessed using the Shapiro–Wilk test. Differences in surface roughness (Ra) and Vickers microhardness (HV) among the investigated materials were analyzed using one-way analysis of variance (ANOVA), followed by Tukey’s post hoc test for pairwise comparisons. For Gram-positive and Gram-negative bacterial strains, a two-way ANOVA was performed to evaluate the effects of material type and microbial strain, as well as their interaction, on the reduction in biofilm biomass. For *Candida albicans*, differences among the tested materials were assessed using one-way ANOVA. Tukey’s post hoc test was applied where appropriate. Statistical significance was established at *p* < 0.05.

## 3. Results

The results of the multidimensional characterization of the investigated flowable resin-based composites are summarized in the following sections. Surface roughness, microhardness, and surface morphology were evaluated to provide an integrated characterization of the investigated flowable resin composites. Overall, measurable differences were observed among the tested materials with respect to their surface properties and their susceptibility to microbial colonization.

### 3.1. Surface Roughness Analysis

Surface roughness measurements revealed differences among the investigated flowable resin-based composites. Overall, all materials exhibited relatively low arithmetic average roughness (Ra) values following the standardized polishing procedure. The lowest mean roughness was observed for G-ænial™ Universal Injectable (0.04 ± 0.01 μm), indicating a relatively homogeneous and smooth surface. BRILLIANT Flow also demonstrated low roughness values (0.09 ± 0.02 μm) with limited variability among specimens.

**Figure 1:**
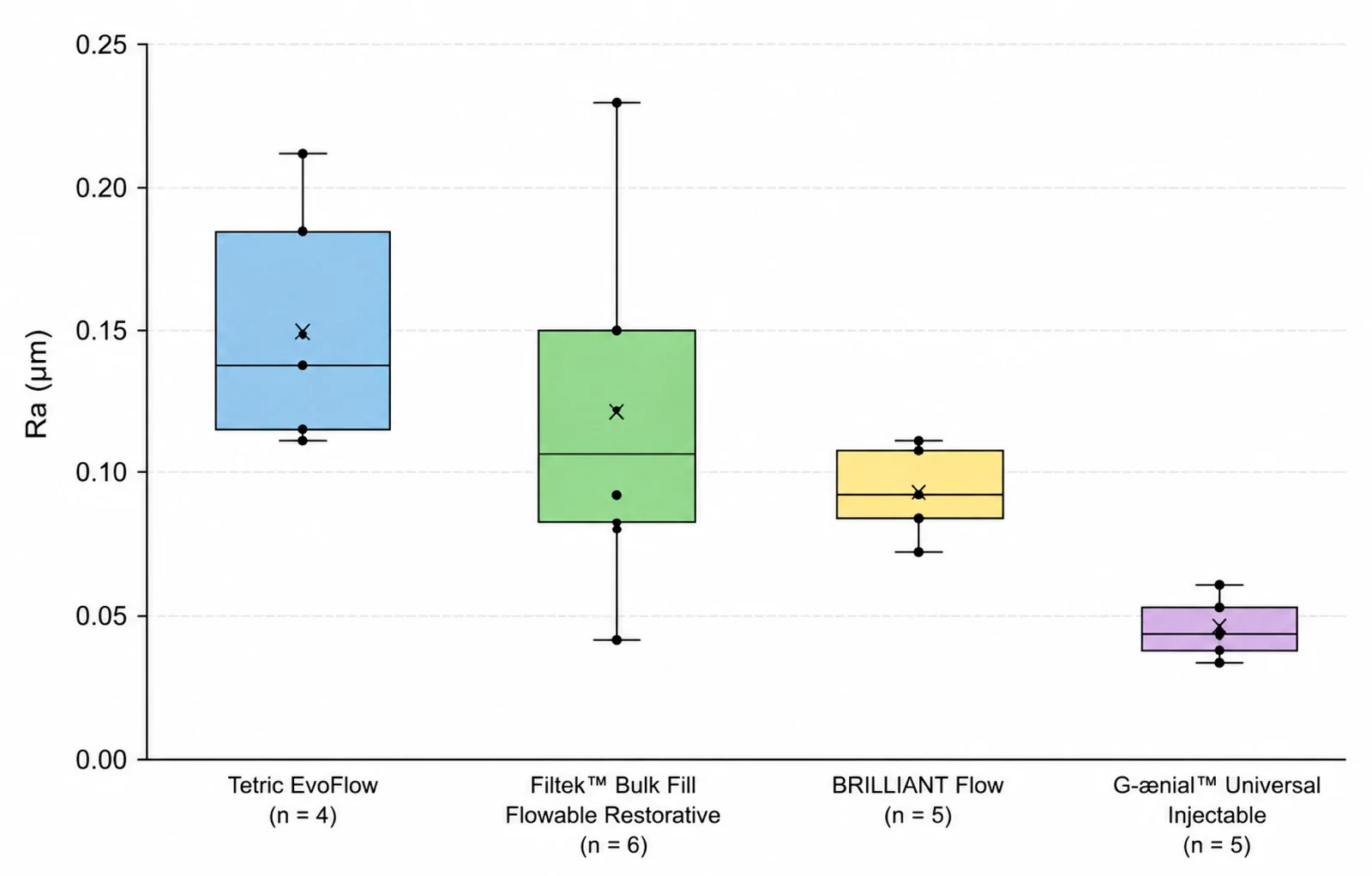
Surface roughness (Ra, μm) of the investigated flowable resin-based composites. Dots represent individual measurements, and × indicates the mean.

Filtek™ Bulk Fill Flowable Restorative exhibited intermediate roughness values (0.12 ± 0.07 μm), with individual measurements ranging from 0.04 μm to 0.23 μm. Among the investigated materials, Tetric EvoFlow showed the highest mean roughness (0.14 ± 0.05 μm), with measurements ranging between 0.11 μm and 0.21 μm, indicating greater variability in surface topography compared with the other composites (Figure 1).

Despite these differences, all tested materials exhibited roughness values below the threshold generally considered critical for bacterial retention. Nevertheless, variations in roughness distribution suggest that differences in filler architecture, resin matrix composition, and polishability may influence the final surface characteristics of the restorative materials.

### 3.2. Vickers Microhardness Analysis

**Figure 2:**
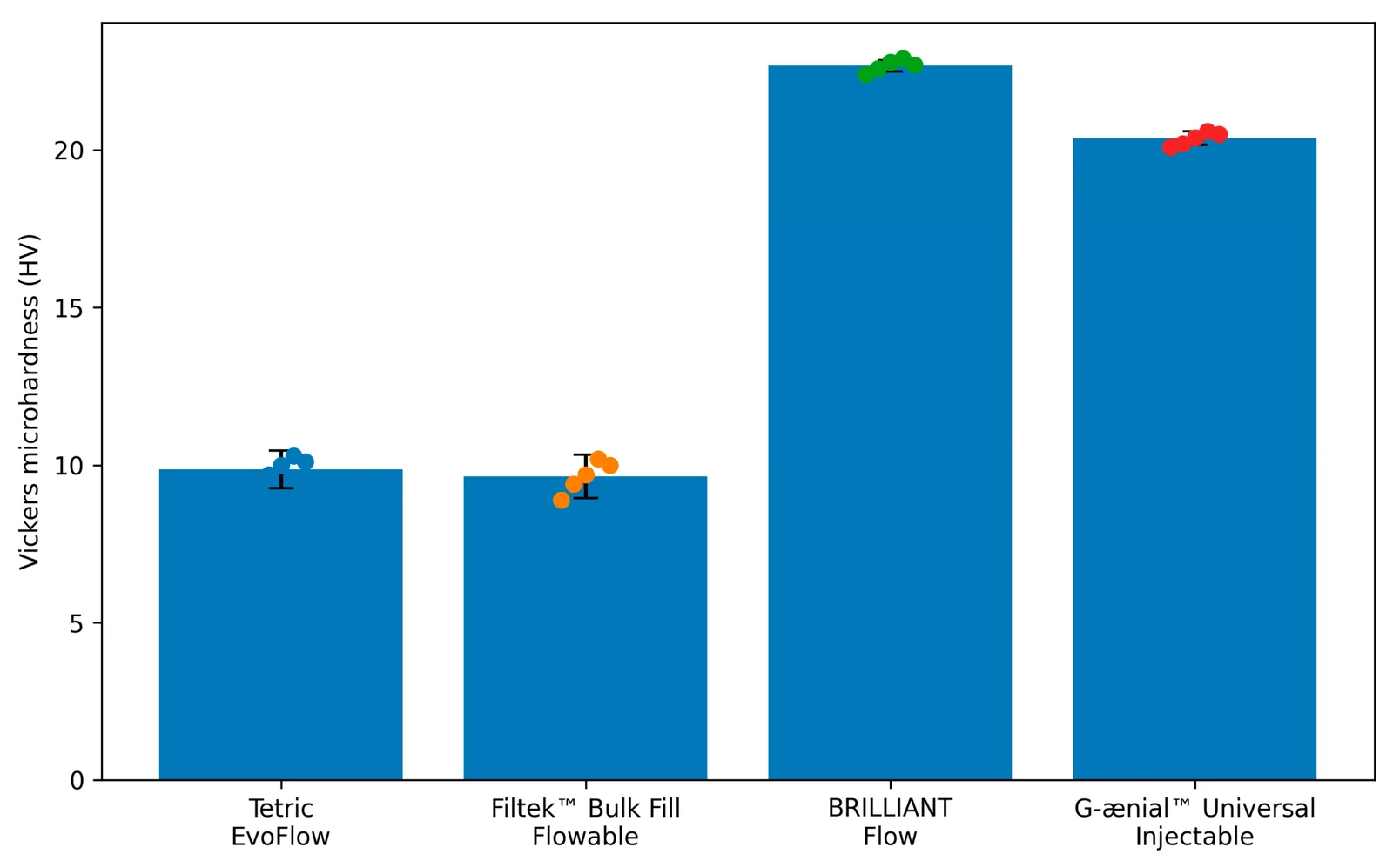
Vickers microhardness (HV) values of the investigated flowable resin-based composites. Colored points represent individual measurements, and bars represent mean values.

The Vickers microhardness values of the investigated flowable resin-based composites are presented in Figure 2. Noticeable differences were observed among the tested materials. BRILLIANT Flow exhibited the highest mean microhardness value (22.68 ± 0.18 HV), followed by G-ænial™ Universal Injectable (20.38 ± 0.22 HV). In contrast, substantially lower hardness values were recorded for Tetric EvoFlow (9.86 ± 0.59 HV) and Filtek™ Bulk Fill Flowable Restorative (9.64 ± 0.69 HV).

Overall, two distinct groups could be identified based on surface hardness. BRILLIANT Flow and G-ænial™ Universal Injectable demonstrated considerably higher hardness values and lower variability among specimens, whereas Tetric EvoFlow and Filtek™ Bulk Fill Flowable Restorative exhibited lower hardness values and greater dispersion. These differences may be attributed to variations in filler loading, filler characteristics, and resin matrix composition.

The relatively low standard deviation values indicate good reproducibility of the measurements and suggest a homogeneous response within each material group. The observed differences in surface hardness provide additional information regarding the mechanical behavior of the investigated composites and may contribute to the interpretation of their surface morphology and microbial biofilm formation behavior.

### 3.3. Surface Morphology and Microstructural Characteristics

**Figure 3:**
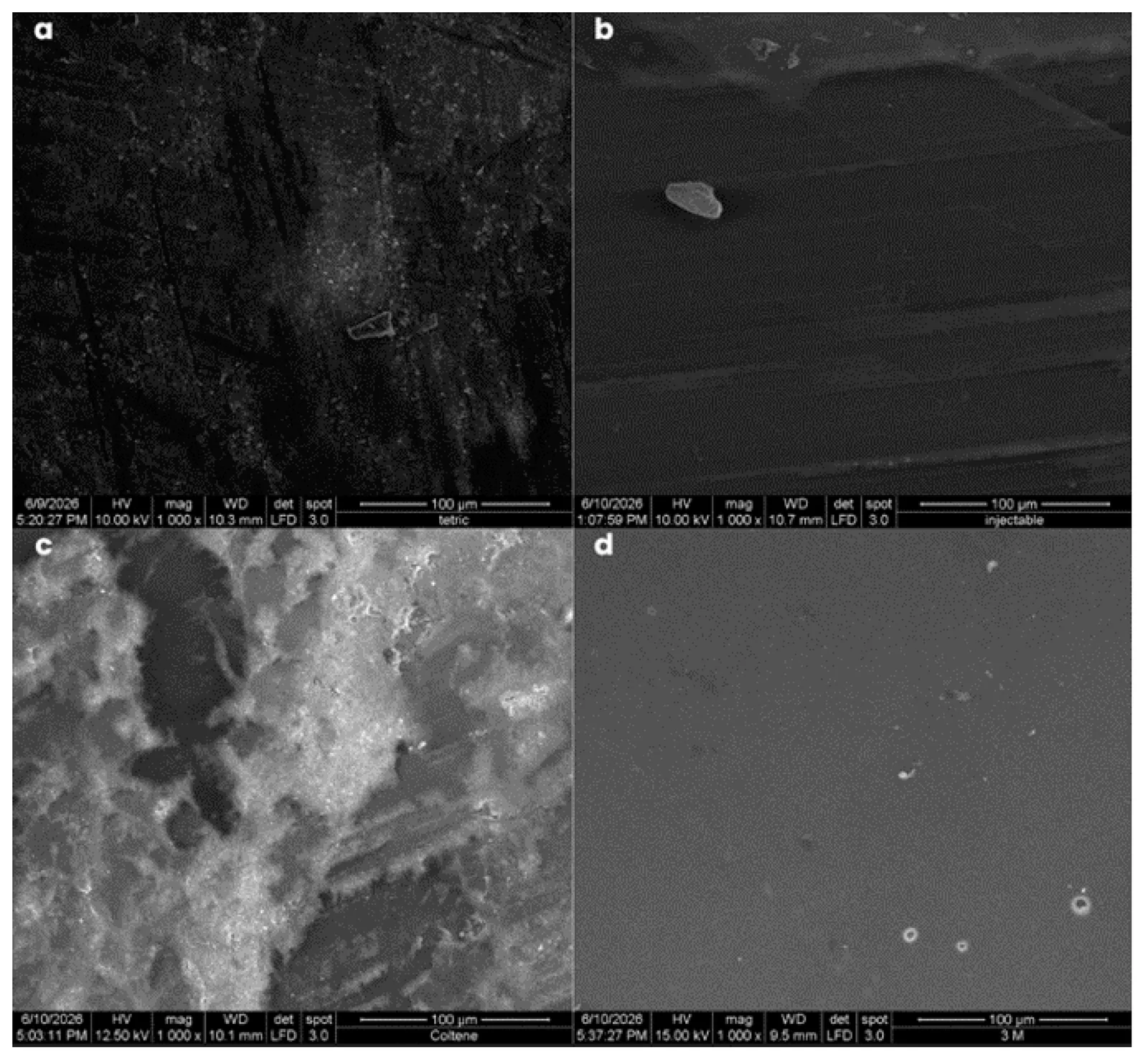
Representative SEM micrographs of the investigated flowable resin-based composites obtained at ×1000 magnification. (**a**) Tetric EvoFlow; (**b**) G-ænial™ Universal Injectable; (**c**) BRILLIANT Flow; and (**d**) Filtek™ Bulk Fill Flowable Restorative.

**Figure 4:**
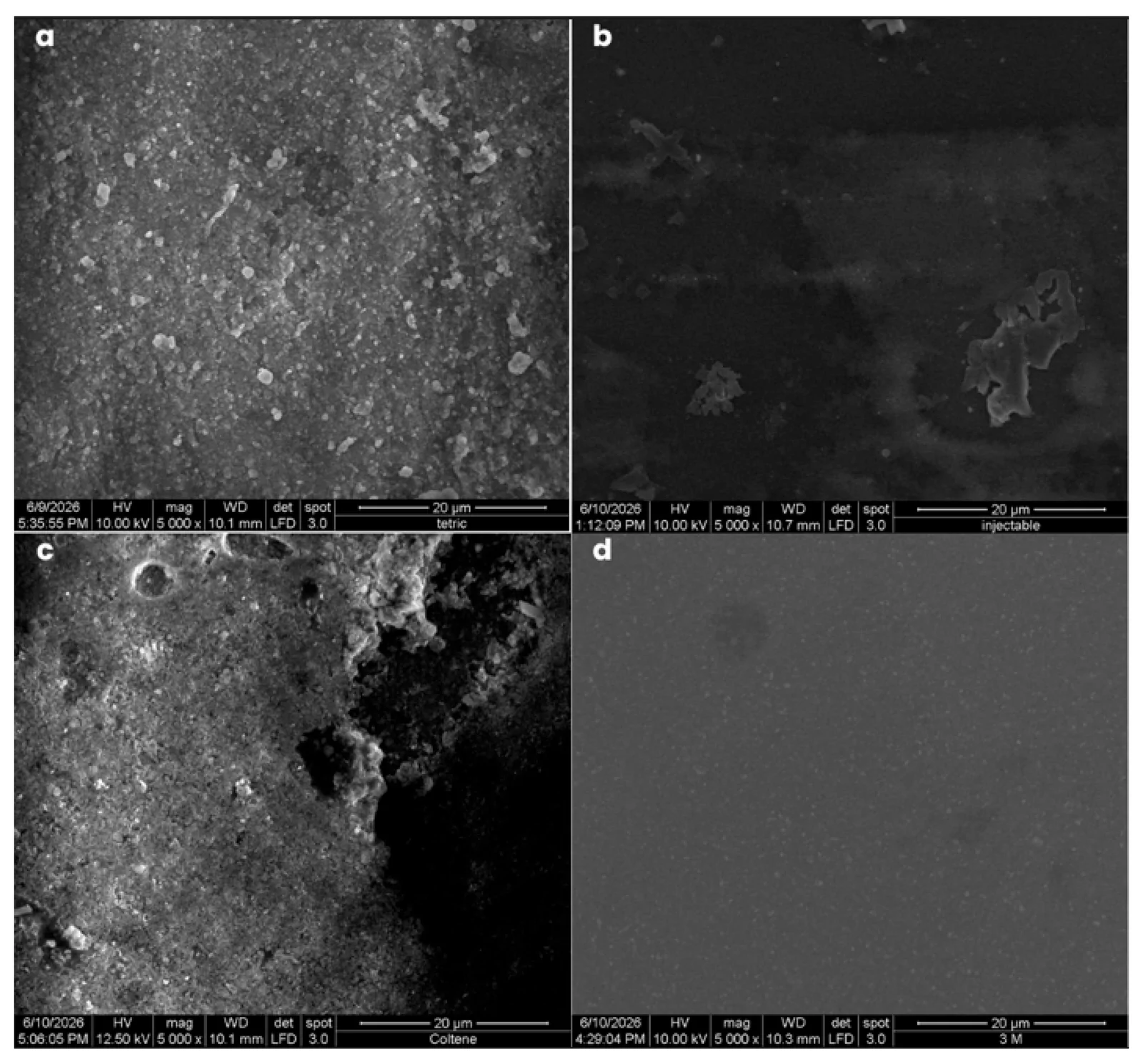
Representative SEM micrographs obtained at ×5000 magnification. (**a**) Tetric EvoFlow; (**b**) G-ænial™ Universal Injectable; (**c**) BRILLIANT Flow; and (**d**) Filtek™ Bulk Fill Flowable Restorative.

**Figure 5:**
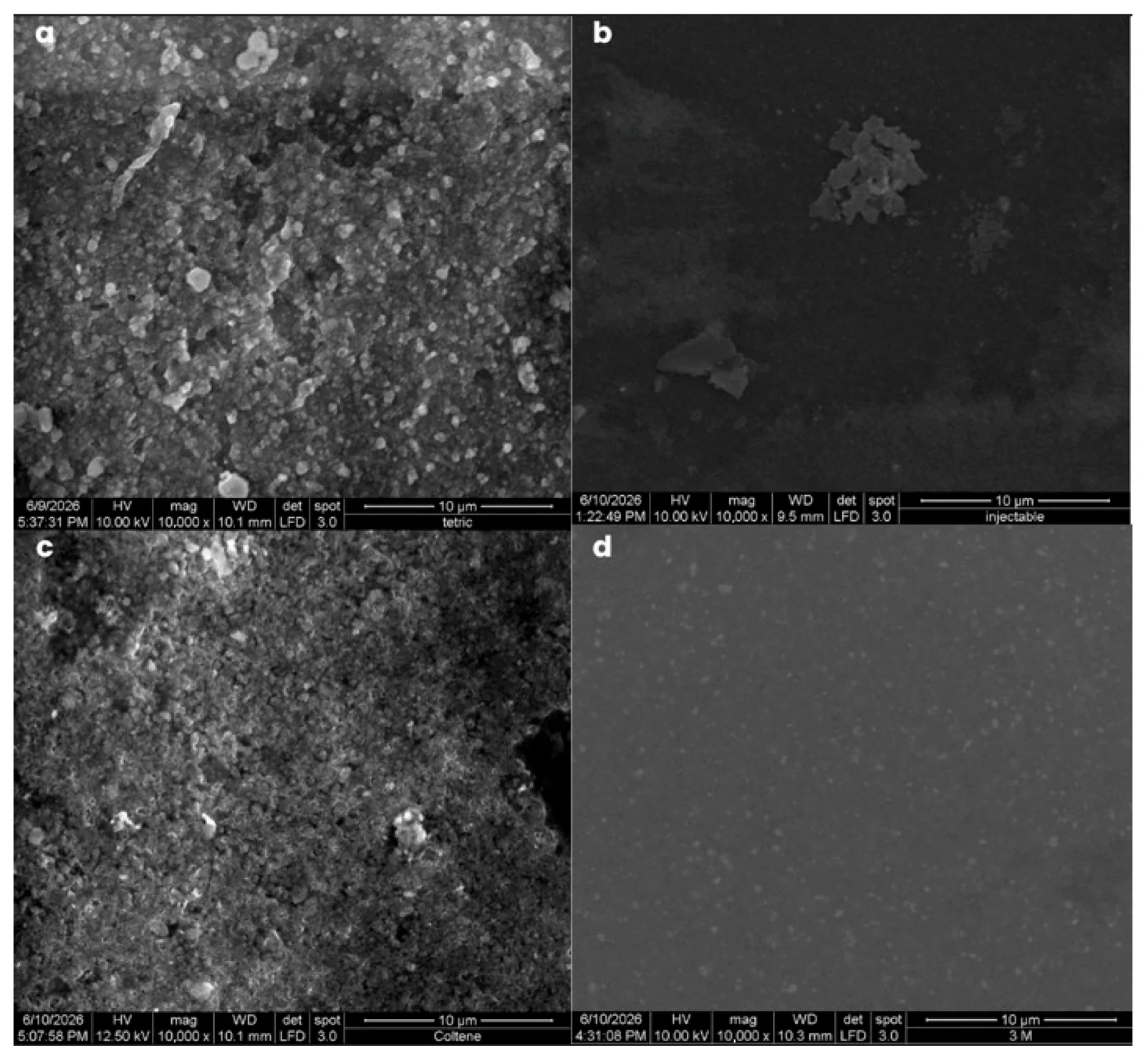
Representative SEM micrographs obtained at ×10,000 magnification. (**a**) Tetric EvoFlow; (**b**) G-ænial™ Universal Injectable; (**c**) BRILLIANT Flow; and (**d**) Filtek™ Bulk Fill Flowable Restorative.

**Figure 6:**
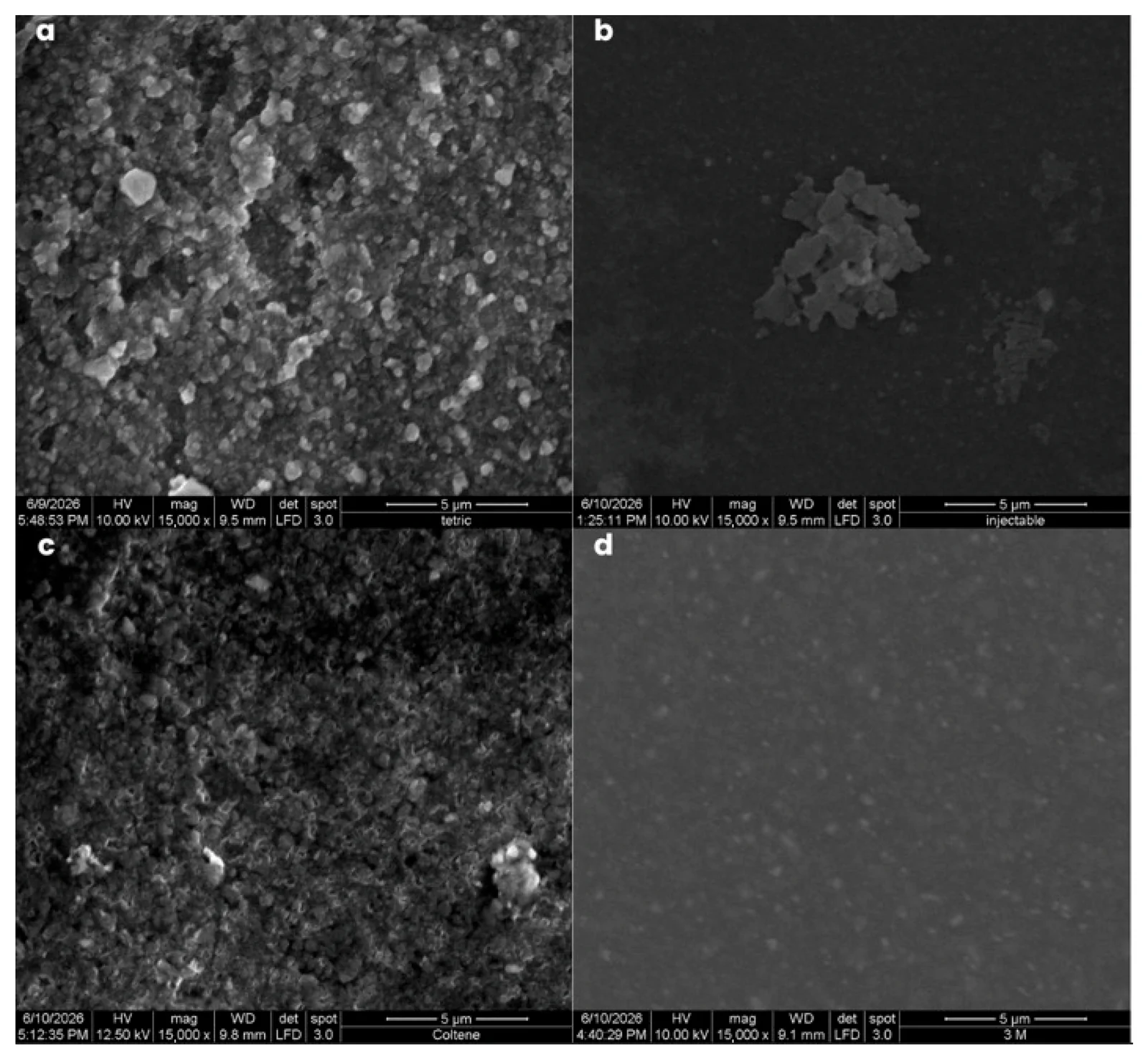
Representative SEM micrographs obtained at ×15,000 magnification. (**a**) Tetric EvoFlow; (**b**) G-ænial™ Universal Injectable; (**c**) BRILLIANT Flow; and (**d**) Filtek™ Bulk Fill Flowable Restorative.

Representative SEM micrographs obtained at different magnifications are presented in Figure 3, Figure 4, Figure 5 and Figure 6. Surface morphology varied among the investigated flowable resin-based composites, revealing differences in surface homogeneity, topographical irregularities, and filler exposure patterns.

At low magnification (×1000, Figure 3), Filtek™ Bulk Fill Flowable Restorative exhibited the smoothest and most homogeneous surface appearance, characterized by minimal irregularities and isolated defects. G-ænial™ Universal Injectable also showed a relatively uniform morphology, although scattered particles and occasional surface imperfections were observed. In contrast, Tetric EvoFlow displayed visible polishing grooves and surface discontinuities, whereas BRILLIANT Flow demonstrated the most heterogeneous morphology, with irregular regions and evident porosities.

At intermediate magnifications (×5000 and ×10,000, Figure 4 and Figure 5), the heterogeneous morphology of Tetric EvoFlow and BRILLIANT Flow became more apparent. Tetric EvoFlow exhibited numerous protruding particles and non-uniform topographical features, while BRILLIANT Flow showed irregular areas and structures suggestive of particle agglomeration. Conversely, Filtek™ Bulk Fill Flowable Restorative maintained a compact and relatively homogeneous appearance, and G-ænial™ Universal Injectable exhibited a smooth matrix with limited surface defects.

At high magnification (×15,000, Figure 6), microstructural differences among materials became more evident. Tetric EvoFlow showed pronounced filler exposure and an irregular surface pattern, whereas BRILLIANT Flow presented discontinuities and heterogeneous regions. In contrast, Filtek™ Bulk Fill Flowable Restorative exhibited a fine and homogeneous microstructure, while G-ænial™ Universal Injectable maintained a relatively smooth surface with fewer detectable defects.

Overall, SEM examination revealed distinct differences in surface morphology among the investigated materials. Filtek™ Bulk Fill Flowable Restorative and G-ænial™ Universal Injectable generally exhibited smoother and more homogeneous surfaces, whereas Tetric EvoFlow and BRILLIANT Flow presented increased topographical heterogeneity and more pronounced surface irregularities. These observations are in agreement with the profilometric findings and provide additional insight into the relationship between surface morphology and the mechanical behavior of the investigated composites.

### 3.4. Integrated Comparative Analysis of Physicochemical Surface Characteristics and Microbial Biofilm Formation

To facilitate an integrated interpretation of the multidimensional characterization performed in the present study, the physicochemical surface properties of the investigated flowable resin composites were comparatively evaluated together with their microbial biofilm formation profiles. The comparative summary of the principal physicochemical parameters and the overall relative biofilm biomass reduction is presented in Table 2.

Although clear differences were identified in surface roughness, Vickers microhardness, and surface morphology among the investigated materials, these variations were not consistently accompanied by proportional differences in microbial biofilm formation. G-ænial™ Universal Injectable exhibited the lowest mean surface roughness, whereas BRILLIANT Flow demonstrated the highest Vickers microhardness. Nevertheless, neither material consistently showed the lowest biofilm formation across all tested microorganisms. Conversely, Tetric EvoFlow displayed the highest surface roughness while exhibiting increased biofilm formation for several Gram-positive strains but not for Gram-negative bacteria or *Candida albicans*.

Overall, the integrated comparison showed no consistent pattern indicating that lower surface roughness or higher microhardness was associated with greater reductions in relative biofilm biomass across the investigated materials. For example, Tetric EvoFlow presented the highest overall relative biofilm biomass reduction despite exhibiting the highest surface roughness and one of the lowest microhardness values, whereas BRILLIANT Flow exhibited the highest microhardness but the lowest overall relative biofilm biomass reduction. These findings indicate that biofilm formation on the investigated materials cannot be explained by a single physicochemical surface characteristic alone and is more likely influenced by the combined contribution of multiple material-related characteristics.

Collectively, these findings support the interpretation that microbial biofilm formation on flowable resin-based composites is a multifactorial process influenced by the combined effects of surface morphology, physicochemical properties, and microorganism-specific biological characteristics.

### 3.5. Quantitative Assessment of Microbial Biofilm Formation

The quantitative assessment of microbial biofilm formation on the investigated flowable resin composites is summarized in Table 3. Overall, all materials exhibited measurable differences in the relative biofilm biomass reduction, with values ranging from 4.37% to 12.21%, depending on both the restorative material and the investigated microorganism. Low standard deviation values indicated good reproducibility of the experimental data. Differences in the relative biofilm biomass reduction were observed among Gram-positive bacteria, Gram-negative bacteria, and *Candida albicans*, suggesting that microbial biofilm formation was influenced by both microorganism-specific characteristics and material-related properties.

#### 3.5.1. Relative Biofilm Biomass Reduction in Gram-Positive Bacteria

**Figure 7:**
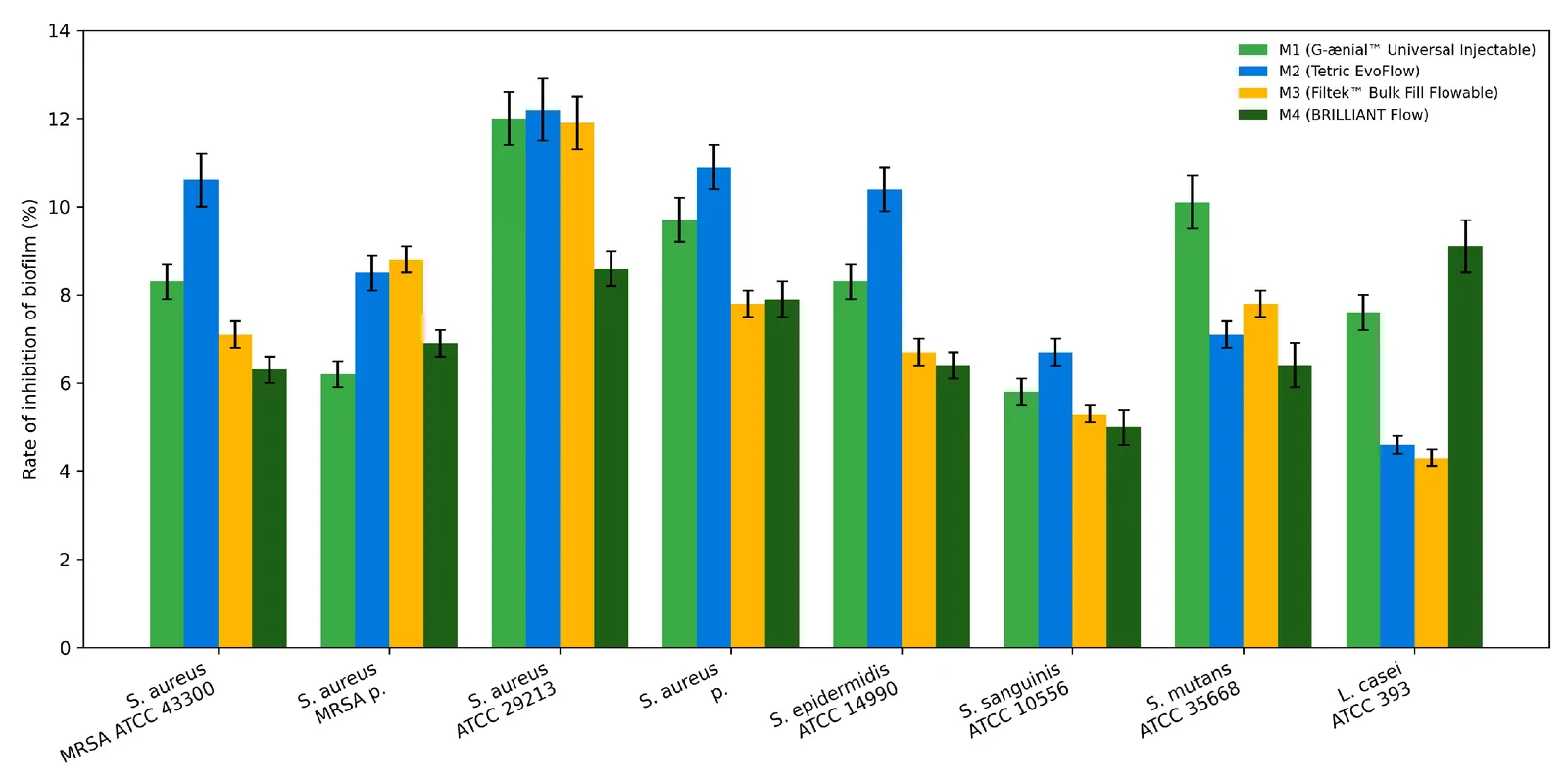
Relative biofilm biomass reduction (%) exhibited by the investigated flowable resin composites against Gram-positive bacterial strains. Data are presented as mean ± SD.

The relative biofilm biomass reduction observed for the investigated flowable resin composites against Gram-positive bacterial strains is presented in Figure 7. Relative biofilm biomass reduction values varied among both the restorative materials and the investigated microorganisms, although the overall differences remained modest. For G-ænial™ Universal Injectable, values ranged from 5.79% for *Streptococcus sanguinis* (ATCC 10556) to 12.07% for *Staphylococcus aureus* (ATCC 29213). Similarly, Tetric EvoFlow exhibited values ranging between 6.66% and 12.21% for the same strains.

In the case of Filtek™ Bulk Fill Flowable Restorative, relative biofilm biomass reduction values ranged from 4.37% for *Lacticaseibacillus casei* (ATCC 393) to 11.90% for *S. aureus* (ATCC 29213), while BRILLIANT Flow showed values between 5.09% and 9.19% depending on the bacterial strain.

Overall, the relative biofilm biomass reduction observed for Gram-positive bacteria remained modest, with only a limited number of measurements exceeding 10%. Differences were identified among the investigated restorative materials as well as among the tested bacterial strains, including both reference strains and clinical isolates. Among the investigated microorganisms, *S. aureus* (ATCC 29213) exhibited the highest relative biofilm biomass reduction, whereas *S. sanguinis* (ATCC 10556) and *L. casei* (ATCC 393) demonstrated the lowest values.

Two-way ANOVA revealed significant effects of both material type and bacterial strain on relative biofilm biomass reduction (*p* < 0.05), as well as a significant interaction between these factors. Although statistically significant differences were identified for several strains, the absolute differences among materials remained relatively small, indicating modest differences in relative biofilm biomass reduction.

**Figure 8:**
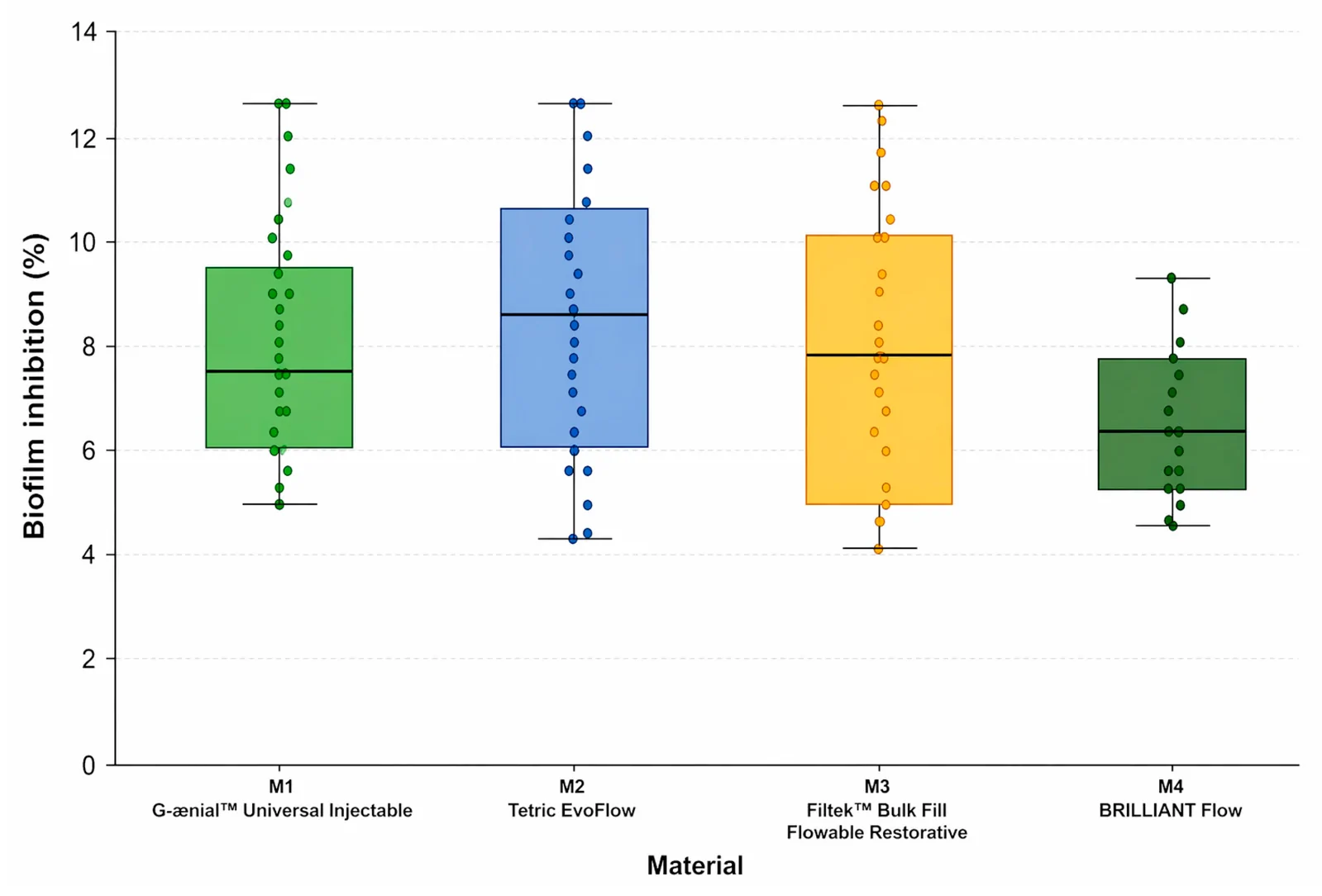
Boxplot representation of relative biofilm biomass reduction values for Gram-positive bacterial strains across the investigated flowable resin composites.

The boxplot representation (Figure 8) illustrates the relatively narrow distribution of relative biofilm biomass reduction values across the investigated materials and confirms the low variability and good reproducibility of the measurements. The tested composites demonstrated limited but measurable but relatively modest differences in relative biofilm biomass reduction against Gram-positive bacteria.

#### 3.5.2. Relative Biofilm Biomass Reduction in Gram-Negative Bacteria

**Figure 9:**
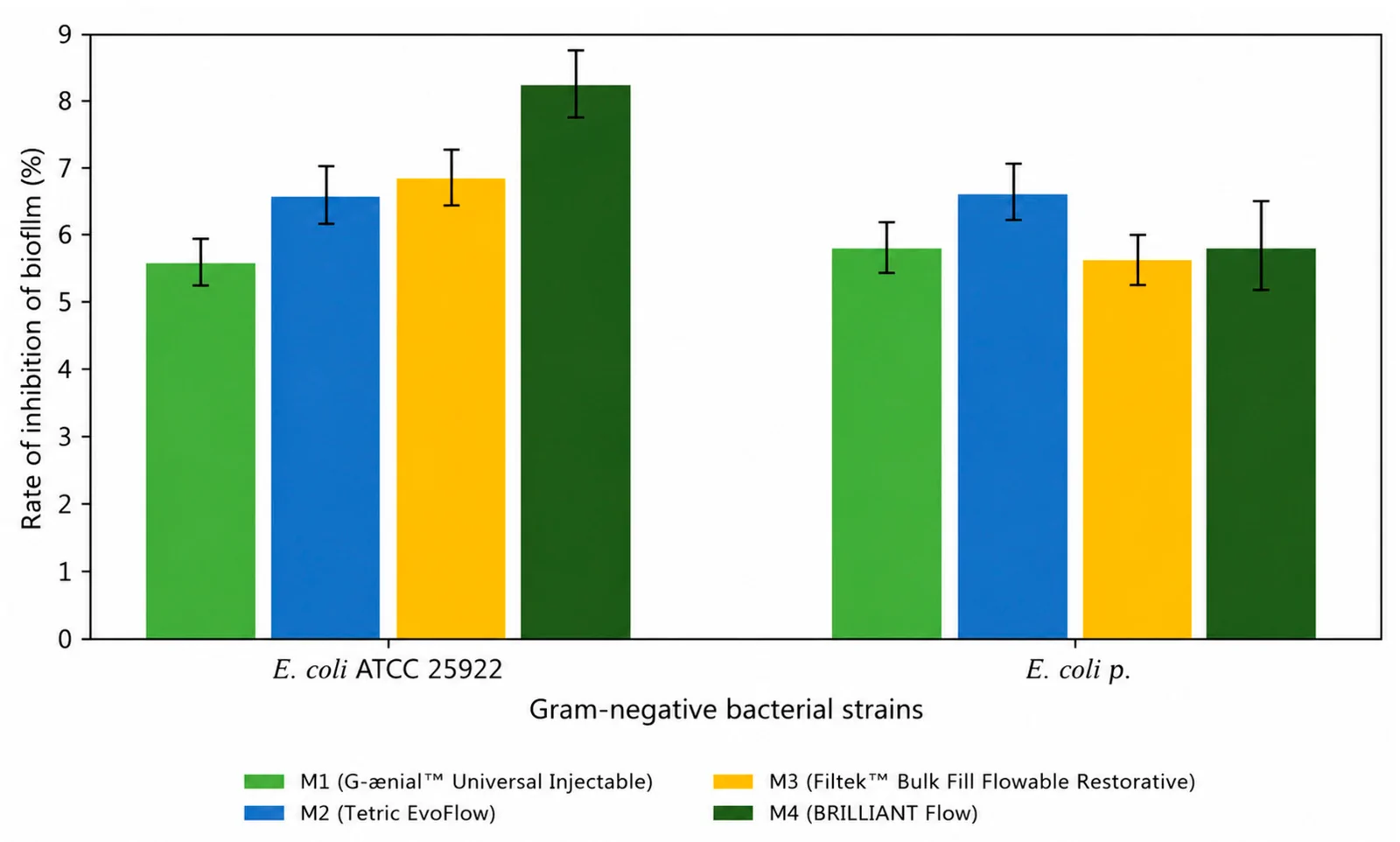
Relative biofilm biomass reduction (%) exhibited by the investigated flowable resin-based composites against Gram-negative bacterial strains. Data are presented as mean ± SD.

The relative biofilm biomass reduction observed for the investigated flowable resin composites on Gram-negative bacterial strains is illustrated in Figure 9. Relative biofilm biomass reduction values remained below 10% for all tested restorative materials and bacterial strains and varied according to both the restorative material and the investigated microorganism.

For G-ænial™ Universal Injectable and Tetric EvoFlow, the highest relative biofilm biomass reduction values were recorded for the clinical isolate of *Escherichia coli*, reaching 5.82% and 6.60%, respectively. In contrast, Filtek™ Bulk Fill Flowable Restorative and BRILLIANT Flow exhibited their highest relative biofilm biomass reduction values for *E. coli* (ATCC 25922), with values of 6.83% and 8.24%, respectively. Across all experimental conditions, the relative biofilm biomass reduction values were relatively close, indicating limited variability among the investigated restorative materials and bacterial strains. Overall, the differences observed between the reference strains and clinical isolates were modest.

Statistical analysis demonstrated a significant overall effect of material type on relative biofilm biomass reduction for Gram-negative strains, as well as a significant effect of strain type and of the material × strain interaction. Two-way ANOVA showed significant differences for material (*p* < 0.001), strain (*p* < 0.001), and their interaction (*p* < 0.001). When the two strains were analyzed separately, one-way ANOVA revealed statistically significant differences among materials for *E. coli* ATCC 25922 (*p* = 0.0005), whereas no significant differences were observed for the clinical *E. coli* isolate (*p* = 0.113). These findings indicate that the microbial biofilm response of Gram-negative bacteria depended not only on the tested composite, but also on the specific bacterial strain. Despite the statistically significant effects detected by two-way ANOVA, the absolute differences between materials remained relatively small, indicating modest differences in relative biofilm biomass reduction among the investigated flowable resin composites.

**Figure 10:**
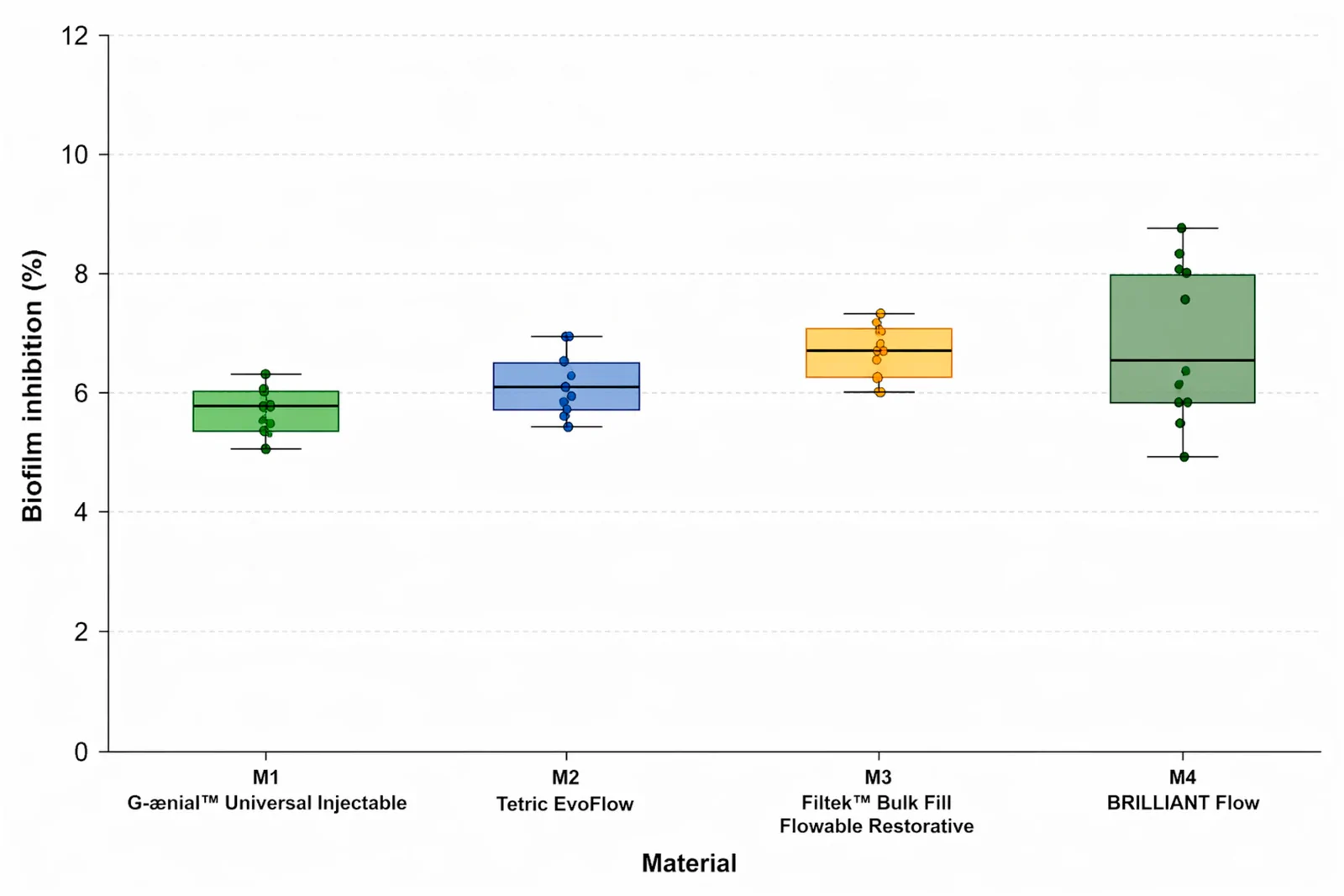
Boxplot representation of relative biofilm biomass reduction values (%) for Gram-negative bacterial strains across the investigated flowable resin composites.

The boxplot representation (Figure 10) illustrates the distribution of relative biofilm biomass reduction values across the investigated materials and highlights a more pronounced separation between groups for *E. coli* ATCC 25922 compared with the clinical isolate. The relatively low variability among measurements further supports the reproducibility of the experimental data.

#### 3.5.3. Relative Biofilm Biomass Reduction in *Candida albicans*

**Figure 11:**
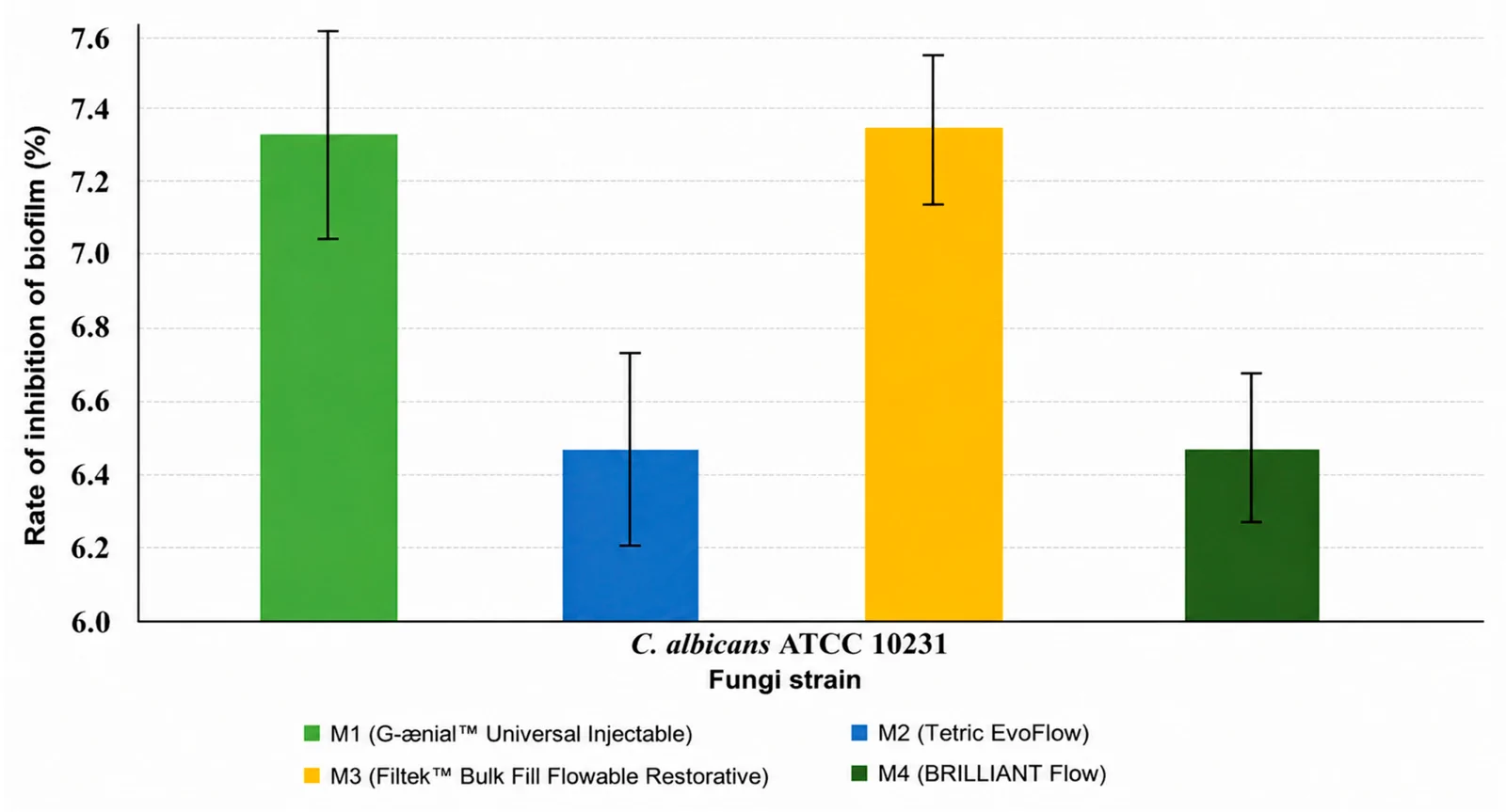
Relative biofilm biomass reduction (%) exhibited by the investigated flowable resin composites against *Candida albicans* (ATCC 10231). Data are presented as mean ± SD.

The relative biofilm biomass reduction observed for the investigated flowable resin composites against the fungal strain *Candida albicans* (ATCC 10231) is presented in Figure 11. The relative biofilm biomass reduction values exhibited a narrow range across the investigated materials, indicating relatively low variability between the experimental groups.

The highest relative biofilm biomass reduction value was recorded for Filtek™ Bulk Fill Flowable Restorative, reaching 7.37%, while the lowest value was observed for BRILLIANT Flow, at 6.46%. Intermediate values were obtained for G-ænial™ Universal Injectable and Tetric EvoFlow, both exhibiting relative biofilm biomass reduction values within the same range. Overall, the differences in relative biofilm biomass reduction values between the tested materials were relatively small, indicating comparable microbial biofilm responses toward *Candida albicans*.

Statistical analysis using one-way ANOVA indicated that the differences between the tested materials were not statistically significant (*p* > 0.05). This finding confirms the relatively homogeneous microbial biofilm response observed across all experimental groups. The low standard deviation values further indicate good reproducibility of the experimental data and minimal variability between replicates. Although minor variations in relative biofilm biomass reduction values were observed, none of the investigated materials demonstrated a statistically superior relative biofilm biomass reduction against *Candida albicans*. Overall, all composites exhibited comparable patterns of relative biofilm biomass reduction.

#### 3.5.4. Comparative Overview of Relative Biofilm Biomass Reduction

**Figure 12:**
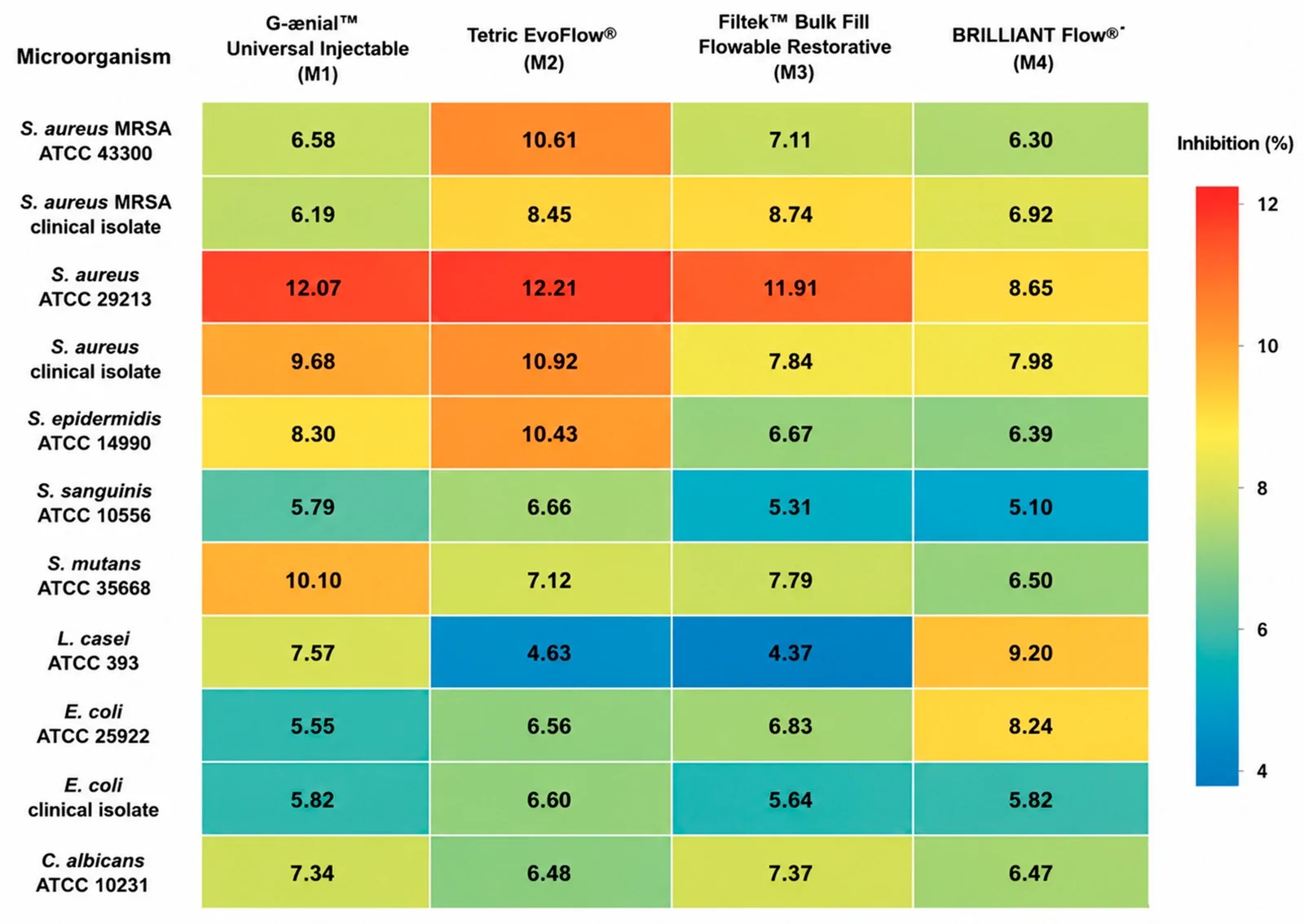
Heatmap representation of relative biofilm biomass reduction (%) exhibited by the investigated flowable resin composites against the tested microorganisms. Color intensity increases from blue (lower relative biofilm biomass reduction) to red (higher relative biofilm biomass reduction).

To facilitate the visualization of relative biofilm biomass reduction patterns across the investigated microorganisms, a heatmap representation was generated (Figure 12). The color distribution highlights the differences in relative biofilm biomass reduction values among the investigated flowable resin-based composites and microbial strains. Overall, the heatmap confirms the relative biofilm biomass reduction remained within a relatively narrow range across all tested materials, with values generally ranging between 4% and 12%.

Higher relative biofilm biomass reduction values were mainly observed against Gram-positive bacteria, particularly *Staphylococcus aureus* (ATCC 29213), whereas lower relative biofilm biomass reduction values were recorded for *Streptococcus sanguinis* (ATCC 10556), *Lacticaseibacillus casei* (ATCC 393), and Gram-negative strains. Tetric EvoFlow exhibited comparatively higher relative biofilm biomass reduction values against several Gram-positive strains, while BRILLIANT Flow demonstrated increased relative biofilm biomass reduction against *L. casei* and *Escherichia coli* (ATCC 25922). The responses observed for *Candida albicans* were relatively homogeneous across all investigated materials.

Overall, the heatmap provides a comprehensive visualization of the relative biofilm biomass reduction patterns and further illustrates that the investigated flowable resin composites exhibited only modest differences in microbial biofilm formation across the tested microorganisms.

## 4. Discussion

The present study provided a multidimensional characterization of four commercially available flowable resin-based composites by combining surface roughness analysis, Vickers microhardness testing, scanning electron microscopy (SEM), and quantitative biofilm biomass assessment. Distinct differences in surface morphology and mechanical behavior were observed among the investigated materials, whereas only modest differences in microbial biofilm biomass accumulation were detected under standardized in vitro conditions. Collectively, these findings provide a comparative characterization of material–biofilm interactions in commercially available flowable resin-based composites.

These findings are consistent with previous reports indicating that conventional resin-based composites generally exhibit comparable patterns of microbial biofilm formation, with material-dependent differences being primarily associated with variations in physicochemical surface characteristics rather than intrinsic antibacterial functionality [15]. Although statistically significant differences were observed among certain materials and microbial strains, their magnitude remained relatively small, suggesting that microbial colonization of restorative materials is governed by multiple interacting physicochemical and biological factors. Overall, the integrated comparison indicated that differences in surface roughness and microhardness were not consistently accompanied by proportional differences in microbial biofilm formation, suggesting that microbial colonization is influenced by multiple interacting physicochemical and biological factors rather than by a single surface characteristic.

In the case of Gram-positive bacteria, slightly higher relative biofilm biomass reduction values were observed compared to Gram-negative strains. This finding may be explained by structural differences in the bacterial cell envelope. Gram-positive bacteria, such as *Streptococcus mutans* and *Staphylococcus aureus*, possess a thick peptidoglycan layer and lack an outer membrane, which may facilitate more direct interaction with the material surface and influence adhesion processes [7].

By contrast, Gram-negative bacteria exhibited lower and more homogeneous relative biofilm biomass reduction values. This behavior can be attributed to the presence of an outer membrane rich in lipopolysaccharides, which acts as a protective barrier and may reduce the influence of surface-related material characteristics on microbial adhesion and early biofilm formation [27]. Similar differences between Gram-positive and Gram-negative microorganisms have been reported in previous studies investigating the interaction between dental materials and oral biofilms [7].

Regarding the fungal strain *Candida albicans*, the relative biofilm biomass reduction values were relatively uniform across all tested materials, suggesting comparable microbial biofilm formation patterns. This finding may be explained by the well-documented capacity of *C. albicans* to form highly organized biofilms surrounded by a dense extracellular matrix, which contributes to increased resistance to antimicrobial agents and environmental stressors [16,28]. Despite the differences observed in surface roughness, microhardness, and microstructural morphology, none of the investigated materials demonstrated consistently lower biofilm biomass accumulation than the others. This finding suggests that microbial interactions with resin-based composites are governed by multiple factors and cannot be explained solely by surface topography or mechanical properties.

The relatively modest differences in relative biofilm biomass reduction observed in the present study may also be explained by the intrinsic resistance mechanisms associated with mature biofilms. It has been well documented that microbial biofilms are highly structured communities embedded within an extracellular polymeric matrix, which limits the penetration of antimicrobial agents and protects microbial cells from both host immune responses and external stressors. In addition, bacteria within biofilms exhibit altered metabolic states and reduced growth rates, further decreasing their susceptibility to conventional antimicrobial approaches [4].

In addition to microbial resistance mechanisms, the interaction between restorative materials and biofilm formation is influenced by surface-related characteristics, including roughness, surface energy, wettability, and microstructural organization. Previous studies have demonstrated that increased surface irregularities may facilitate microbial retention and biofilm maturation, thereby contributing to plaque accumulation at restoration margins. Moreover, both surface roughness and wettability have been shown to significantly affect multispecies biofilm adhesion and microbial colonization on restorative materials, highlighting the importance of surface properties in material-biofilm interactions [29]. These observations are consistent with previous evidence [14], suggesting that biofilm formation on resin-based composites reflects the complex interplay between material physicochemical characteristics and microorganism-specific biological features rather than the influence of any individual surface property.

The differences observed among the investigated materials may be attributed to variations in resin matrix composition, filler architecture, particle size distribution, and filler loading. These compositional factors are known to influence surface topography, mechanical behavior, and microbial biofilm formation. It has been demonstrated that dental restorative materials can serve as substrates for microbial adhesion and biofilm development, depending on their physicochemical properties [25,29].

In the present study, profilometric and SEM analyses revealed distinct differences in surface morphology among the investigated composites. G-ænial™ Universal Injectable exhibited the lowest surface roughness values, whereas Tetric EvoFlow showed greater variability in surface topography. SEM examination further demonstrated that Filtek™ Bulk Fill Flowable Restorative presented the most homogeneous surface morphology, while Tetric EvoFlow and BRILLIANT Flow exhibited increased heterogeneity and localized surface irregularities. Despite these differences, no material consistently exhibited lower biofilm biomass accumulation than the others, suggesting that microbial adhesion and biofilm development are influenced by multiple factors beyond surface topography alone.

From a clinical perspective, surface smoothness, mechanical stability, and resistance to microbial colonization are important determinants of the long-term success of restorative procedures. Surface-related characteristics, including filler type, particle size, resin matrix composition, and roughness, influence microbial adhesion and biofilm development, while persistent biofilms at restoration margins may contribute to discoloration, marginal degradation, and secondary caries. These observations are consistent with previous studies highlighting the relationship between surface characteristics and biofilm formation on resin-based composites [30].

Recent advances in biomaterials research have shown that modifications in filler composition and the incorporation of bioactive components may enhance the biological functionality of resin-based systems, supporting the development of multifunctional restorative materials capable of interacting with the oral environment [2].

In this context, the slightly higher relative biofilm biomass reduction values observed for materials such as Tetric EvoFlow and Filtek™ Bulk Fill Flowable Restorative may be associated with differences in filler architecture and resin formulation, which can influence surface energy, wettability, and microbial adhesion. Nevertheless, the relatively small differences observed among the investigated materials indicate that biofilm formation cannot be attributed to any single material characteristic but rather reflects the combined influence of multiple surface-related and microorganism-specific factors.

From a clinical perspective, the relatively modest differences in relative biofilm biomass reduction observed for all investigated materials suggests that restoration longevity depends not only on the intrinsic properties of the composite, but also on patient-related factors such as oral hygiene, dietary habits, and salivary composition. Effective plaque control and preventive measures therefore remain essential for minimizing biofilm accumulation and reducing the risk of secondary caries. These findings are particularly relevant for patients with elevated caries risk, in whom individualized preventive strategies and risk-based treatment approaches remain crucial for long-term restorative success [31,32]. Accordingly, the present findings should be interpreted as comparative laboratory observations rather than direct evidence supporting clinical material selection. Nevertheless, standardized comparative laboratory characterization of commercially available restorative materials provides an important experimental reference framework for the objective evaluation and future development of advanced multifunctional restorative biomaterials under identical testing conditions.

Despite these observations, no consistent correspondence was identified between surface roughness, microhardness, and relative biofilm biomass reduction, supporting the interpretation that biofilm formation results from the combined influence of multiple material-related and microorganism-specific factors rather than from any single physicochemical property. Although G-ænial™ Universal Injectable exhibited the lowest roughness values and Filtek™ Bulk Fill Flowable Restorative showed the most homogeneous surface morphology under SEM examination, neither material demonstrated consistently lower microbial biofilm biomass accumulation. Conversely, Tetric EvoFlow exhibited comparatively higher relative biofilm biomass reduction values despite presenting greater surface heterogeneity.

Recent research has focused on the development of advanced antimicrobial dental materials, incorporating agents such as quaternary ammonium compounds, silver nanoparticles, or calcium phosphate fillers, aiming to enhance their biological performance [7,33]. These strategies include both controlled-release systems and contact-active materials designed to inhibit bacterial adhesion and viability. Recent studies have demonstrated that the incorporation of drug-loaded nanocarriers, such as chlorhexidine-loaded mesoporous silica nanoparticles, may significantly improve resistance to microbial biofilm formation through sustained-release mechanisms, overcoming the limitations of conventional composites that rely mainly on passive surface interactions [2]. The findings of the present study are consistent with previous research highlighting the predominantly passive biological behavior of conventional resin-based composites. Although the incorporation of antimicrobial agents can improve antibacterial performance, maintaining prolonged and controlled release remains a major challenge [8].

These approaches support the transition from passive restorative materials toward multifunctional systems capable of modulating the oral microbiome and providing additional therapeutic benefits. In addition, smart or stimuli-responsive biomaterials have emerged as promising candidates for next-generation restorative systems. By responding to environmental changes such as pH variations or enzymatic activity, these materials may provide targeted antimicrobial effects and improved biological performance, representing a shift from passive restorative materials toward active and responsive biomaterials [34]. In this context, the modest differences in relative biofilm biomass reduction observed in the present study further emphasize the need for the development of multifunctional restorative materials capable of combining favorable mechanical properties with improved biological functionality.

Despite these advances, the present findings further emphasize the complexity of material–biofilm interactions in conventional flowable resin-based composites, highlighting the need for continued optimization through multifunctional and bioactive approaches. Additionally, the inclusion of both reference strains and clinical isolates provides a more realistic evaluation of microbial behavior, as clinical isolates often exhibit increased variability and resistance compared to laboratory strains [25]. Importantly, the overall findings indicate that no single physicochemical surface characteristic adequately explained the observed microbial biofilm formation patterns, further supporting the concept that biofilm development on restorative materials is a multifactorial process influenced by the combined effects of surface morphology, physicochemical properties, and microbial characteristics. These findings emphasize the importance of integrated material characterization rather than relying on individual physicochemical parameters when evaluating material–biofilm interactions. From a biomaterials perspective, the results highlight the need for the development of multifunctional restorative materials capable of combining favorable mechanical properties with improved biofilm-modulating properties. Strategies such as the incorporation of bioactive fillers, antimicrobial agents, or contact-active surfaces represent promising directions for enhancing the biological functionality of restorative composites. Such approaches may contribute to improving restoration longevity and reducing the risk of biofilm-related complications. Although modest differences in relative biofilm biomass were observed among the investigated materials, these findings should be interpreted with caution. The crystal violet assay employed in the present study provides a quantitative estimate of total adhered biofilm biomass but does not distinguish viable from non-viable microorganisms, assess metabolic activity, or separately quantify microbial cells and extracellular polymeric matrix. Therefore, differences in biofilm biomass alone cannot be considered direct evidence of biologically or clinically meaningful antibiofilm efficacy. Instead, the present findings contribute to the comparative characterization of material–biofilm interactions under standardized laboratory conditions. Their biological and clinical relevance should be confirmed through complementary viability and metabolic assays, as well as progressively more complex experimental models and clinical investigations [35,36,37,38].

The present experimental design represents a deliberately standardized laboratory model rather than an attempt to reproduce the biological complexity of the oral ecosystem. While saliva conditioning, multispecies microbial interactions, anaerobic conditions, and dynamic biofilm maturation undoubtedly influence biofilm development in vivo, simplified in vitro models remain an essential component of biomaterials research because they enable reproducible comparisons under controlled conditions. Recent methodological reviews further emphasize that no single biofilm model can fully replicate the oral environment and that model selection should be guided by the specific research objective, with progressively more complex multispecies, saliva-conditioned, aging, and dynamic systems representing subsequent stages of biomaterial validation [35,37,39,40].

However, several limitations should be considered when interpreting the present findings. The microbial panel employed in this study was intentionally selected to provide a standardized comparative evaluation of material–biofilm interactions under controlled laboratory conditions rather than to reproduce the full microbiological complexity of secondary caries or mature oral biofilms. The relative biofilm biomass reduction was evaluated under in vitro conditions, which may not fully replicate the complexity of the oral environment, including salivary flow, pellicle formation, and multispecies biofilms. Furthermore, the investigated microorganisms were evaluated individually, whereas natural oral biofilms are polymicrobial and involve complex interspecies interactions. In addition, the microbial panel consisted predominantly of aerobic and facultative anaerobic microorganisms and did not include obligate anaerobic species, such as *Porphyromonas gingivalis*, *Fusobacterium nucleatum*, or *Prevotella* spp., which are important components of mature oral biofilms and periodontal infections. Surface roughness, microhardness, and SEM analyses were performed without artificial aging procedures, such as thermocycling, long-term water storage, or mechanical fatigue loading, which may substantially influence the long-term physicochemical and biological behavior of restorative materials in the oral environment [41,42,43]. Therefore, the present findings should be interpreted within the context of a standardized in vitro laboratory model and should not be directly extrapolated to clinical material selection or clinical decision-making. The primary strength of this experimental approach lies in its ability to provide reproducible and objective comparisons among commercially available restorative materials under controlled conditions while minimizing biological variability. As emphasized in recent methodological recommendations, simplified standardized biofilm models represent an essential first step in the sequential evaluation of restorative biomaterials, whereas saliva-conditioned, multispecies, anaerobic, dynamic, and aging models are required for subsequent validation prior to clinical translation. Future studies should therefore incorporate clinically relevant artificial aging procedures, including thermocycling, long-term water storage, and mechanical fatigue loading, together with saliva conditioning, multispecies biofilm models including cariogenic and obligate anaerobic microorganisms, and dynamic experimental systems to better reproduce the oral environment and further validate the present findings.

Future research should focus on the development of multifunctional and bioactive restorative materials with integrated therapeutic potential. The incorporation of antimicrobial agents, bioactive fillers, and stimuli-responsive components may enable the transition from passive restorative systems toward active biomaterials capable of modulating microbial behavior and improving long-term clinical outcomes.

## 5. Conclusions

This study provided a multidimensional characterization of four commercially available flowable resin-based dental composites by combining surface roughness analysis, Vickers microhardness testing, scanning electron microscopy, and quantitative biofilm biomass assessment. Distinct differences in surface morphology and mechanical behavior were observed among the investigated materials, whereas only modest differences in relative biofilm biomass reduction were observed under standardized in vitro conditions.

The findings indicate that microbial biofilm formation on restorative materials is a multifactorial phenomenon that cannot be explained by a single physicochemical surface characteristic. Although differences in surface roughness, microhardness, and microstructure were identified, none of the investigated materials consistently demonstrated reduced biofilm biomass accumulation under the experimental conditions employed. Accordingly, the observed differences in biofilm biomass should be interpreted as comparative laboratory findings rather than evidence of biologically or clinically meaningful antibiofilm efficacy. Within the limitations of this standardized in vitro laboratory model, the present study provides a reproducible comparative evaluation of commercially available flowable resin composites. Accordingly, the findings should not be directly extrapolated to clinical material selection or clinical decision-making.

Future studies should validate these findings using progressively more biologically relevant experimental models and further investigate strategies for developing multifunctional restorative biomaterials with improved biofilm-modulating properties.

## Figures and Tables

**Table 1:** Characteristics of the investigated flowable resin-based dental composites.

Material	Manufacturer (Company, City, Country)	Resin Matrix Composition	Filler Type	Filler Size	Filler Content (wt%/vol%)	Material Classification	Viscosity/Handling	Clinical Indications (Manufacturer)	Batch (Lot No.)
Filtek™ Bulk Fill Flowable Restorative	3M, St. Paul, MN, USA	Bis-GMA, UDMA, Bis-EMA, Procrylat resins	Non-agglomerated silica, zirconia/silica clusters	4–20 nm (silica); up to 10 µm (clusters)	~64.5 wt%/~42.5 vol%	Bulk-fill flowable nanocomposite	Low viscosity (bulk-fill)	Posterior restorations (bulk fill), base/liner	11467702
Tetric EvoFlow	Ivoclar Vivadent, Schaan, Liechtenstein	Bis-GMA, UDMA, D3MA	Barium glass, ytterbium trifluoride, mixed oxides	40–3000 nm	~63 wt%/~40 vol%	Flowable nanohybrid composite	Low viscosity (flowable)	Class I–V restorations, liners, small cavities	Z07VT4
BRILLIANT Flow	Coltene, Altstätten, Switzerland	Bis-GMA, UDMA, TEGDMA	Barium glass, amorphous silica	20–2000 nm	~62 wt%/~38 vol%	Flowable nanohybrid composite	Low viscosity (flowable)	Minimally invasive restorations, fissure sealing	N 51783
G-ænial™ Universal Injectable	GC Corporation, Tokyo, Japan	UDMA, dimethacrylates (proprietary monomers)	Silica, barium glass, pre-polymerized fillers	~200 nm + pre-polymerized particles (µm range)	~69 wt%/~50 vol%	Injectable nanohybrid composite	Injectable, highly flowable	Universal restorations, cervical lesions, liners	240516A

**Table 2:** Integrated comparative summary of physicochemical surface characteristics and overall relative biofilm biomass reduction in the investigated flowable resin composites.

Material	Surface Roughness (Ra, μm) Mean ± SD	Vickers Microhardness (HV) Mean ± SD	Overall Relative Biofilm Biomass Reduction (%)
G-ænial™ Universal Injectable	0.04 ± 0.01	20.38 ± 0.22	7.73
Tetric EvoFlow	0.14 ± 0.05	9.86 ± 0.59	8.24
Filtek™ Bulk Fill Flowable Restorative	0.12 ± 0.07	9.64 ± 0.69	7.23
BRILLIANT Flow	0.09 ± 0.02	22.68 ± 0.18	7.05

**Table 3:** Relative biofilm biomass reduction (%) expressed as mean ± standard deviation (SD) for the investigated flowable resin composites.

Microbial Strain	M1 (G-ænial™) Mean ± SD	M2 (Tetric EvoFlow) Mean ± SD	M3 (Filtek™ Bulk Fill Flowable Restorative) Mean ± SD	M4 (BRILLIANT Flow) Mean ± SD
*S. aureus* MRSA ATCC 43300	6.58 ± 0.40	10.61 ± 0.65	7.11 ± 0.29	6.30 ± 0.25
*S. aureus* MRSA *p.*	6.19 ± 0.31	8.45 ± 0.38	8.74 ± 0.36	6.92 ± 0.37
*S. aureus* ATCC 29213	12.07 ± 0.62	12.21 ± 0.72	11.91 ± 0.68	8.65 ± 0.36
*S. aureus p.*	9.68 ± 0.49	10.92 ± 0.56	7.84 ± 0.34	7.98 ± 0.38
*S. epidermidis* ATCC 14990	8.30 ± 0.38	10.43 ± 0.49	6.67 ± 0.27	6.39 ± 0.37
*S. sanguinis* ATCC 10556	5.79 ± 0.25	6.66 ± 0.28	5.31 ± 0.23	5.10 ± 0.47
*S. mutans* ATCC 35668	10.10 ± 0.66	7.12 ± 0.31	7.79 ± 0.34	6.50 ± 0.51
*L. casei* ATCC 393	7.57 ± 0.38	4.63 ± 0.22	4.37 ± 0.22	9.20 ± 0.57
*E. coli* ATCC 25922	5.55 ± 0.29	6.56 ± 0.30	6.83 ± 0.39	8.24 ± 0.49
*E. coli p.*	5.82 ± 0.27	6.60 ± 0.31	5.64 ± 0.25	5.82 ± 0.56
*C. albicans* ATCC 10231	7.34 ± 0.35	6.48 ± 0.25	7.37 ± 0.28	6.47 ± 0.22

## Data Availability

The data presented in this study are available on request from the corresponding authors.

## Abbreviations

The following abbreviations are used in this manuscript:

ANOVA	Analysis of Variance
ATCC	American Type Culture Collection
Bis-EMA	Ethoxylated Bisphenol A Dimethacrylate
Bis-GMA	Bisphenol A Glycidyl Methacrylate
*C. albicans*	*Candida albicans*
CFU	Colony-Forming Units
CHX	Chlorhexidine
CHX-MSN	Chlorhexidine-Loaded Mesoporous Silica Nanoparticles
D3MA	Decanediol Dimethacrylate
*E. coli*	*Escherichia coli*
EPS	Extracellular Polymeric Substance
HV	Vickers Hardness Number
MRSA	Methicillin-Resistant *Staphylococcus aureus*
OD	Optical Density
p	Probability Value
Ra	Arithmetic Average Surface Roughness
*S. aureus*	*Staphylococcus aureus*
*S. epidermidis*	*Staphylococcus epidermidis*
*S. mutans*	*Streptococcus mutans*
*S. sanguinis*	*Streptococcus sanguinis*
SD	Standard Deviation
SEM	Scanning Electron Microscopy
SPSS	Statistical Package for the Social Sciences
TEGDMA	Triethylene Glycol Dimethacrylate
TSB	Tryptic Soy Broth
UDMA	Urethane Dimethacrylate
wt%	Weight Percentage
vol%	Volume Percentage
μm	Micrometer
°C	Degrees Celsius
